# The RNA helicase DHX34 functions as a scaffold for SMG1-mediated UPF1 phosphorylation

**DOI:** 10.1038/ncomms10585

**Published:** 2016-02-04

**Authors:** Roberto Melero, Nele Hug, Andrés López-Perrote, Akio Yamashita, Javier F. Cáceres, Oscar Llorca

**Affiliations:** 1 Centro de Investigaciones Biológicas (CIB), Consejo Superior de Investigaciones Científicas (Spanish National Research Council, CSIC), Ramiro de Maeztu 9, Madrid 28040, Spain; 2 MRC Human Genetics Unit, Institute of Genetics and Molecular Medicine, Western General Hospital, University of Edinburgh, Edinburgh EH4 2XU, UK; 3 Department of Molecular Biology, Yokohama City University School of Medicine, 3-9, Fukuura, Kanazawa-ku, Yokohama, Kanagawa 236-0004, Japan

## Abstract

Nonsense-mediated decay (NMD) is a messenger RNA quality-control pathway triggered by SMG1-mediated phosphorylation of the NMD factor UPF1. In recent times, the RNA helicase DHX34 was found to promote mRNP remodelling, leading to activation of NMD. Here we demonstrate the mechanism by which DHX34 functions in concert with SMG1. DHX34 comprises two distinct structural units, a core that binds UPF1 and a protruding carboxy-terminal domain (CTD) that binds the SMG1 kinase, as shown using truncated forms of DHX34 and electron microscopy of the SMG1–DHX34 complex. Truncation of the DHX34 CTD does not affect binding to UPF1; however, it compromises DHX34 binding to SMG1 to affect UPF1 phosphorylation and hence abrogate NMD. Altogether, these data suggest the existence of a complex comprising SMG1, UPF1 and DHX34, with DHX34 functioning as a scaffold for UPF1 and SMG1. This complex promotes UPF1 phosphorylation leading to functional NMD.

## Abstract (web summary)

UPF1 is a central Nonsense-mediated mRNA decay—(NMD), a mechanism to degrade mRNAs containing premature translation termination codons-factor—whose phosphorylation is key to triggering NMD. Here the authors show that the DHX34 helicase acts as a scaffold in promoting UPF1 phosphorylation by SMG1 to promotes NMD.

## Graphical abstract

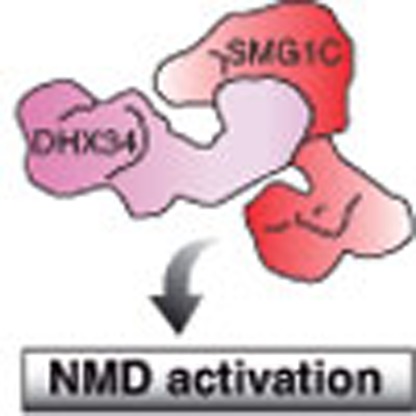

Nonsense-mediated mRNA decay (NMD) is a quality-control mechanism that removes mRNAs containing premature termination codons (PTCs)^1^,^2^,^3^. NMD also plays a more general role in regulating gene expression by controlling decay of a significant fraction of mRNAs in eukaryotes^4^. Recent evidence has revealed that NMD is critical for stem cell differentiation^5^,^6^.

In mammals, initiation of NMD is triggered by the assembly of large complexes containing several up-frameshift (UPF) factors, UPF1, UPF2 and UPF3, bound to the target mRNA^2^,^7^. UPF1 is a 130-kDa RNA helicase composed of two recombinase A (RecA)-like domains at its C terminus and an N-terminal regulatory domain^8^,^9^. In its closed conformation, an N-terminal cysteine–histidine-rich domain packs against two (RecA)-like domains to inhibit the ATPase/helicase activity^8^. UPF1 catalytic activity is regulated by UPF2 (ref. 10), which binds the cysteine–histidine-rich domain and induces a large conformation change that removes the inhibition of the ATPase activity^9^. In higher eukaryotes, a C-terminal domain in UPF1 contributes to its own regulation, apparently in a UPF2- and UPF3-independent manner^11^. UPF1 is a highly processive RNA helicase and its ATPase activity is required to disassemble messenger ribonucleoproteins undergoing NMD^11^,^12^.

Phosphorylation of UPF1 by the SMG1 kinase at several sites in both N- and C-terminal disordered tails of UPF1 is a major event determining the activation of mRNA degradation^2^,^3^,^13^,^14^ and phosphorylated UPF1 is one of the first cellular markers for an NMD target^15^. Therefore, understanding the molecular mechanisms that regulate SMG1-mediated UPF1 phosphorylation is essential to comprehend how the NMD pathway discriminates between normal and aberrant translation termination. Of note, SMG1 is not present in all eukaryotes (yeast lacks SMG1, for instance)^14^.

SMG1 is a large protein (410 kDa) that belongs to the phosphoinositol 3-kinase-related kinase (PIKK) family. The N terminus in all PIKKs is made of a long stretch of helical repeats, mostly HEAT (Huntington, elongation factor 3, a subunit of PP2A and TOR1) repeats. The C terminus comprises three main conserved regions known as a FAT (FRAP, ATM and TRRAP) domain, followed by a catalytic domain with homology to PI3 kinases (PIKK domain hereafter) and ending in a short C-terminal region named FATC^16^. The sequence of SMG1 shows a large insertion after the kinase domain, of unclear structure and function^17^. High-resolution structural information of the conserved region at the C terminus of the PIKK family is provided by atomic structures of the C-terminal region of mammalian target of rapamycin (mTOR), a member of the PIKK family. These showed that the FAT domain consist mostly of α-helices wrapping around the catalytic domain^18^. On the other hand, a 6.6-Å resolution crystal structure of full-length DNA-PKcs showed that the HEAT repeat regions form helical scaffolds^19^. The structural organization of SMG1 has been recently defined at 17–20 Å resolution by single-particle electron microscopy (EM) showing that the conserved C terminus forms a compact globular region (the ‘head') from which the helical N-terminal regions protrude (the ‘arm')^20^,^21^. A model for the architecture of SMG1 was proposed by fitting the atomic structure of mTOR^18^ at the ‘head' and a fragment of DNA-PKcs crystal structure^19^ at the ‘tail' of the EM density for SMG1, and several domains were tentatively localized^21^.

The kinase activity of SMG1 is downregulated by SMG8 (991 amino acids) and SMG9 (520 amino acids)^22^,^23^,^24^. Structures (17–20 Å resolution) of SMG1 and the SMG1–SMG8–SMG9 complex (named SMG1C for ‘SMG1C complex') obtained using EM have revealed that an SMG8–SMG9 complex binds to the SMG1 N-terminal regions inducing a large conformational change^20^,^21^. It is not entirely clear how the kinase activity is regulated by these interactions. In this regard, it was recently shown that SMG8 and SMG9 interact with the SMG1-specific C-terminal insertion, to promote high-affinity binding to UPF1 (ref. 20). Furthermore, UPF2 and UPF3 can activate SMG1 kinase activity *in vivo*, although mammalian NMD events that do not require the intervention of UPF2 and/or UPF3 have also been described^25^,^26^,^27^,^28^. Recent EM structures of SMG1C–UPF1 complexes revealed that UPF1 binds SMG1 at the proximity of its putative kinase domain^20^,^21^. At 17–20 Å resolution, these structures were unable to define the exact position of SMG1 kinase domain, but nonetheless they revealed UPF1, the substrate of the kinase, attached to the ‘head', possibly mapping to the vicinity of the kinase domain. The attachment of UPF1 to SMG1C revealed substantially conformational flexibility that could be stabilized using mild cross-linking^21^.

Additional *trans*-acting factors have been identified using a variety of strategies, such as proteomic approaches or genome-wide RNA interference screens^29^,^30^,^31^; however, in most cases, their mechanism of action remain to be elucidated. In particular, recent additions have enlarged the list of proteins that contribute to regulate UPF1 phosphorylation and NMD, including RuvBL1 and RuvBL2, two ATPases of the AAA+ family^32^,^33^, and DHX34 (DEAH box protein 34), an RNA helicase of the SF2 superfamily^31^,^34^. These proteins have been found to interact with components of the NMD machinery and they seem to promote molecular transitions that are important to activate NMD.

DHX34 is an RNA helicase of the DEAH box family^35^, comprising several domains commonly found in this subfamily of ATPases. The helicase core of DEAH box proteins is formed by two (RecA)-like domains, a winged-helix domain and a helical bundle domain, known as the Ratchet domain^36^. In addition, DEAH box proteins have an auxiliary accessory C-terminal OB (oligonucleotide/oligosaccharide-binding fold) domain (Fig. 1a), which can regulate conformational changes in the DEAH box helicases^36^,^37^. DHX34 associates with several NMD factors in cell lysates, preferentially binding to hypophosphorylated UPF1 (ref. 38). DHX34 contributes to activate UPF1 phosphorylation, but the molecular mechanism for this remains obscure. Current evidence suggests that DHX34 promotes changes in the pattern of interactions between NMD factors that typically associate with NMD activation^38^.

Here we reveal that DHX34 functions as a scaffold to recruit UPF1 to SMG1. A specialized C-terminal domain in DHX34 binds to SMG1 but, importantly, UPF1- and SMG1-recruiting sites are not mutually exclusive, thus allowing the assembly of a tripartite complex containing SMG1, UPF1 and DHX34. The direct binding of DHX34 to the SMG1 kinase through its C-terminal domain promotes UPF1 phosphorylation, leading to functional NMD.

## Results

### 3D architecture of DHX34

**Figure 1:**
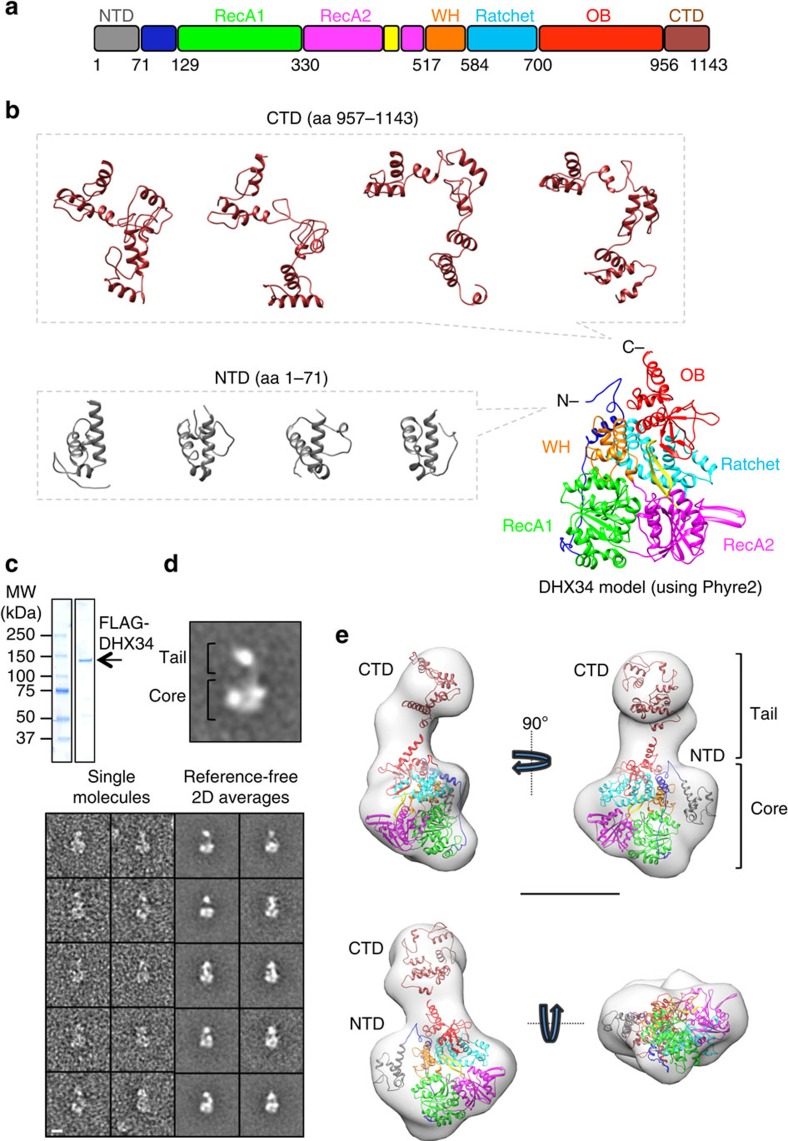
Architecture of DHX34 helicase. (**a**) Cartoon depicting the functional domains of DHX34, showing residue numbers that define their boundaries. Names for domains are borrowed from the structure of Prp43 (ref. 40, 41) and based on the predictions obtained using PHYRE2 (ref. 39). NTD, RecA1, RecA2, winged-helix (WH), Ratchet, OB-fold and CTD domains are shown. The RecA2 domain contains a small antiparallel β-hairpin shown in yellow. (**b**) Atomic modelling of DHX34 obtained using PHYRE2 (ref. 39), including the low-confidence predictions for the NTD and CTD. (**c**) SDS–PAGE (4–15%) of purified FLAG-DHX34 used for the structural analysis. One microgram of FLAG-DHX34 was loaded and stained with SimplyBlue SafeStain (Novex). (**d**) Gallery of selected single molecules of DHX34 observed using EM, as well as reference-free two-dimensional (2D) averages. Scale bar, 10 nm. One representative average has been amplified, and the Tail and Core regions indicated. (**e**) Four views of the 24-Å resolution EM structure of DHX34, shown as a transparent density, where the atomic predictions have been fitted. Scale bar, 5 nm.

Human DHX34 is a DEAH-box RNA helicase containing several domains commonly found in this subfamily of ATPases (Fig. 1a); however, its structure has not yet been defined experimentally. Structure predictions using PHYRE2 (ref. 39) revealed that the core of DHX34 highly resembles yeast Prp43 in complex with ADP (PDB ID 3KX2)^40^, another DEAH-box RNA helicase^41^. The three-dimensional (3D) structure of the DHX34 core, comprising 734 residues and 64% of the total sequence, was predicted with high confidence (residues modelled at 100% confidence), using as template the crystal structure for Prp43 (Fig. 1b and Supplementary Fig. 1a). These results also showed that residues 1–71 and 957–1,143 at the N- and C-terminal ends of the protein (NTD, CTD from now on, respectively) could not be predicted with a significant confidence. In addition, some predictions suggested disorder propensity accumulating in the C-terminal regions of DHX34 and this feature was not so noticeable in other DEAH box helicases^35^ (Supplementary Fig. 1b). *De novo* models for NTD and CTD scored extremely low, exhibiting the absence of any significance in these predictions (Fig. 1b). Nonetheless, these models were informative of the general size and shape that could be perhaps expected for these regions at low resolution and several models for each domain were obtained. Whereas NTD models appeared as a small, probably compact, domain, models for CTD showed a potentially more intricate architecture, as an elongated shape containing different degrees of bending in each of the models proposed.

Next, we analysed the overall shape of DHX34 at low resolution by single-particle EM, with the help of staining agents, as proteins this size are currently extremely difficult to study using frozen specimens. For this purpose, transiently expressed FLAG-tagged DHX34 was affinity purified under high stringency conditions from HEK293T cells (Fig. 1c). Images of single molecules were sufficiently clear to reveal that DHX34 was structured in two regions, one compact globular region (Fig. 1d, placed at the bottom of each molecule image; named ‘core' hereafter) and a protrusion (Fig. 1d, placed at the top; named ‘tail' hereafter). A significant fraction of molecule images in the micrographs were larger, consisting of associations of single molecules, and some appeared to represent dimeric species (Supplementary Fig. 2). We believe these larger associations possibly represent a tendency of the protein to aggregate *in vitro*, but we cannot discard a putative functional significance of the larger aggregates, especially for the dimers (Supplementary Fig. 2). Nonetheless, we only found monomeric DHX34 interacting with SMG1 (see below).

The structural organization of DHX34 was further revealed after 17,752 images of single molecules of DHX34 monomers were classified and processed to obtain reference-free averages for homogenous views of the molecule (Fig. 1d). These single-molecule images were used to refine the 3D structure of DHX34 at 25 Å resolution (Fig. 1e and Supplementary Fig. 3). The EM structure showed that DHX34 was organized as a globular core and a tail, and the core was interpreted as corresponding to the helicase part of the protein, as there was a good match between the atomic model of DHX34 and the EM when fitted within the EM density (cross-correlation=0.86) (Fig. 1e). Consequently, the apparent protrusion from the EM structure could only be interpreted as the remaining part of the sequence, the CTD, and several of the atomic predictions for this domain matched reasonably the general shape and dimensions of the protrusion (Fig. 1e brown colour). The NTD was tentatively placed within an available density in the EM structure for DHX34, unoccupied after fitting the predicted atomic model (Fig. 1e grey colour). Such region was proximal to the N-terminal end of the fitted atomic model, suggesting some likelihood for this assumption. Nevertheless, these fitting experiments must not be interpreted as an atomic model of DHX34, due to the resolution provided by these analyses, but only as a way to identify, place and describe major structures features in the architecture of DHX34. This strategy demonstrates that DHX34 is organized in two district structural regions, a globular core containing the helicase domain and the C-terminal protrusion.

### Direct interaction between DHX34 and SMG1

**Figure 2:**
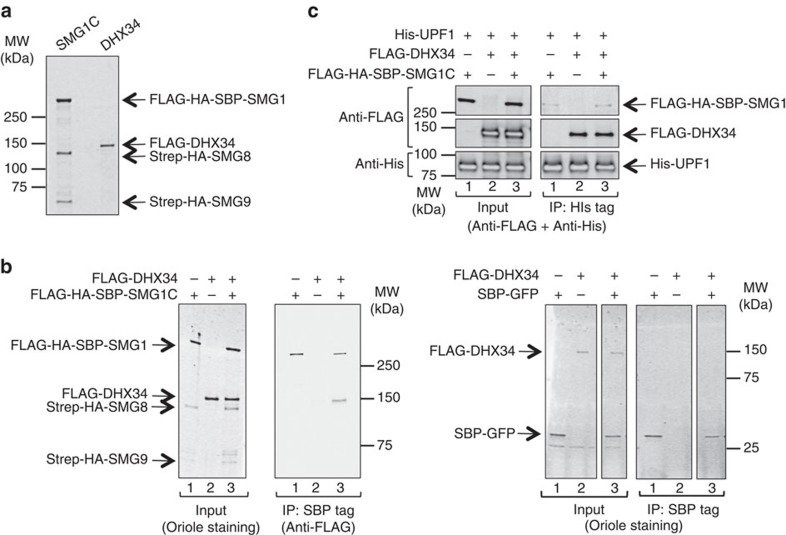
Interactions between SMG1C and DHX34. (**a**) SDS–PAGE of purified FLAG-DHX34 and FLAG-SBP-HA-SMG1C used for structural studies. Purified proteins were resolved in a 4–15% SDS–PAGE and stained using Oriole Fluorescent Gel Stain. (**b**) Pull-down experiment testing the interaction of purified FLAG-DHX34 and FLAG-SBP-HA-SMG1C. Proteins bound to Streptavidin Sepharose beads were eluted using biotin and analysed by SDS–PAGE and western blotting against the FLAG-tag in SMG1 and DHX34. Right panel shows a control experiment demonstrating that DHX34 is not eluted when SBP-GFP is used as bait instead of FLAG-SBP-HA-SMG1C. (**c**) Pull-down experiment testing the interaction of His-UPF1 with FLAG-SBP-HA-SMG1C and FLAG-DHX34. Proteins were mixed and His-UPF1 bound to and eluted from the beads. SDS–PAGE (4–15%) where proteins were identified by western blotting using antibodies against the FLAG and His tags.

DHX34 has been shown to associate to complexes containing several NMD factors, but a direct interaction has only been demonstrated for DHX34 and UPF1 (ref. 38). We have now used purified proteins to determine whether SMG1, DHX34 and UPF1 can interact directly. Recombinant SMG1C was prepared by co-expressing FLAG-HA-SBP-SMG1, Strep-HA-SMG8 and Strep-HA-SMG9, and purified by affinity using the Streptavidin-Binding Peptide (SBP) tag as previously described^21^,^22^ (Fig. 2a). The use of SMG1C was justified, as this complex is more stable for structural studies than SMG1 alone in our hands^22^, and there is no reason to anticipate that SMG8–SMG9 interferes with DHX34 binding. UPF1, lacking the disordered N- and C-terminal tails (residues 115–914) was produced as a His-tag protein as previously described^21^.

FLAG-HA-SBP-SMG1C was incubated with FLAG-DHX34, bound to Streptavidin Sepharose beads and eluted with biotin (Fig. 2b). DHX34 and SMG1C were found in a direct complex as revealed by their co-elution. In contrast, no DHX34 was found in the eluted fractions when SMG1C was absent or substituted by SBP-GFP (Fig. 2b). SMG1C-DHX34 form an abundant complex, based on the relative ratio of the anti-FLAG detection for both proteins (Fig. 2b). As DHX34 also interacts directly with UPF1 (ref. 38), we analysed the interaction of UPF1 with DHX34 and SMG1C using His-UPF1 (Fig. 2c). His-tag pull-down experiments followed by the elution of bound proteins confirmed that UPF1 binds SMG1C and DHX34, as shown before^20^,^21^,^38^.

### Structure of the SMG1C–DHX34 complex

The amount of SMG1C and DHX34 that we can obtain from human cells was largely insufficient to reconstitute the SMG1C–DHX34 complex and purify it by gel filtration chromatography. Instead, we used the power of single-molecule EM and image processing to discern *in silico* those images corresponding to the SMG1C–DHX34 complex.

**Figure 3:**
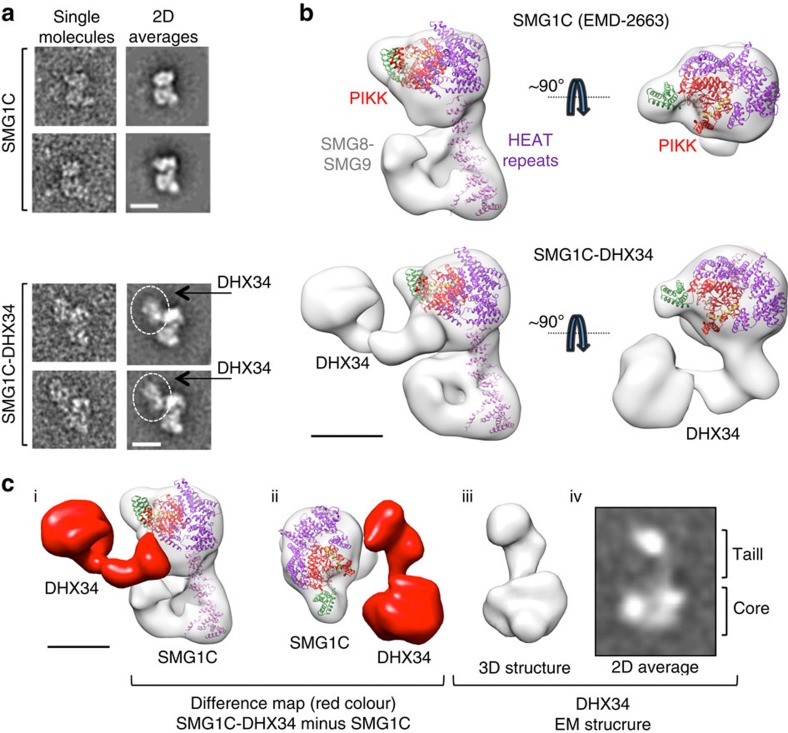
Architecture of the SMG1C–DHX34 complex. (**a**) A gallery of two representative images of SMG1C and SMG1C–DHX34 molecules, and two representative two-dimensional (2D) averages of SMG1C and SMG1C–DHX34. The images revealed SMG1C structured into a compact head (at the top of each molecule in these panels) and a thin arm (at the bottom of each molecule in these panels), similar to those described before^21^. DHX34 interacts with the head region and its location in the images has been highlighted within a circle. Scale bar, 10 nm. (**b**) Two approximately orthogonal views of the EM structures (grey transparent density) of SMG1 (EMD-2663)^21^ (top panels) and SMG1C–DHX34 (bottom panels). A model of SMG1 in each complex was obtained by fitting a HEAT repeat segment of DNA-PKcs (PDB ID 3KGV)^19^ and the catalytic region of mTOR crystal structures (PDB ID 4JSP)^18^, as described before^21^. The putative location of FRB (green), FAT (purple), PIKK domain (red) and HEAT repeats (purple) on that model are indicated, following a previously published model^21^. Scale bar, 5 nm. (**c**) Two views (i,ii) of the difference map obtained after subtracting the structure of SMG1C to SMG1C–DHX34. The difference is shown as a solid red density represented on the structure of the SMG1C used for subtraction, with the atomic model fitted within the EM density^21^. A view of the 3D structure (iii) and 2D average (iv) of DHX34 alone is shown for comparison with the structure of DHX34 in complex with SMG1 (i,ii). Scale bar, 5 nm.

SMG1C and DHX34 were incubated together (1:1.5 SMG1C/DHX34 molar ratio) and the putative complex was chemically fixed for its observation in the electron microscope as described before^21^. Images (60,070) of individual molecules were extracted from the micrographs, processed and classified. An image classification strategy was designed to identify those images from the mixture corresponding to free DHX34 and SMG1C, and those derived from the SMG1C–DHX34 complex (Supplementary Fig. 4). The strategy was based on our knowledge of the images obtained for DHX34 and SMG1C alone, which facilitated finding those where SMG1C was attached to an additional density (Supplementary Fig. 4). A large fraction of images resulting from the incubation of SMG1 and DHX34 were similar to those obtained for each protein alone, but image processing also found 13,080 molecule images, never found in DHX34 or SMG1C, where a density, corresponding to DHX34, was bound to SMG1C (Fig. 3a). The comparison of the averages obtained for SMG1C–DHX34 and SMG1C suggested that DHX34 contacted the head region, where the C terminus of SMG1 has been assigned^21^ (Fig. 3a).

### The CTD domain recruits DHX34 to SMG1

The structure of SMG1C and the structural model published have identified the C terminus of SMG1, comprising the kinase domain, FAT, FATC and FRB as a globular ‘head' region in SMG1 (EMD-2663)^21^ (Fig. 3b). Images of single molecules of the SMG1C–DHX34 complex were used to refine the 3D structure of the complex at 21 Å resolution (Fig. 3b, Supplementary Fig. 5 and Supplementary Movie 1). The density corresponding to DHX34 in SMG1C–DHX34 was identified after alignment and subtraction of the structure of SMG1C from SMG1C–DHX34 (Fig. 3c, i and ii, difference map shown in red colour). The structure of DHX34 bound to SMG1C was very similar to that of DHX34 in isolation (Fig. 3c, iii and iv) and the structure also demonstrated that it was the CTD that interacted with the SMG1 head domain, whereas the helicase core remained unattached to SMG1. Interestingly, DHX34 CTD was found to contact a region that, according to the modelling, corresponds to the vicinities of the kinase domain^21^ (Fig. 3b and Supplementary Movie 1, kinase domain labelled as PIKK and in red colour).

**Figure 4:**
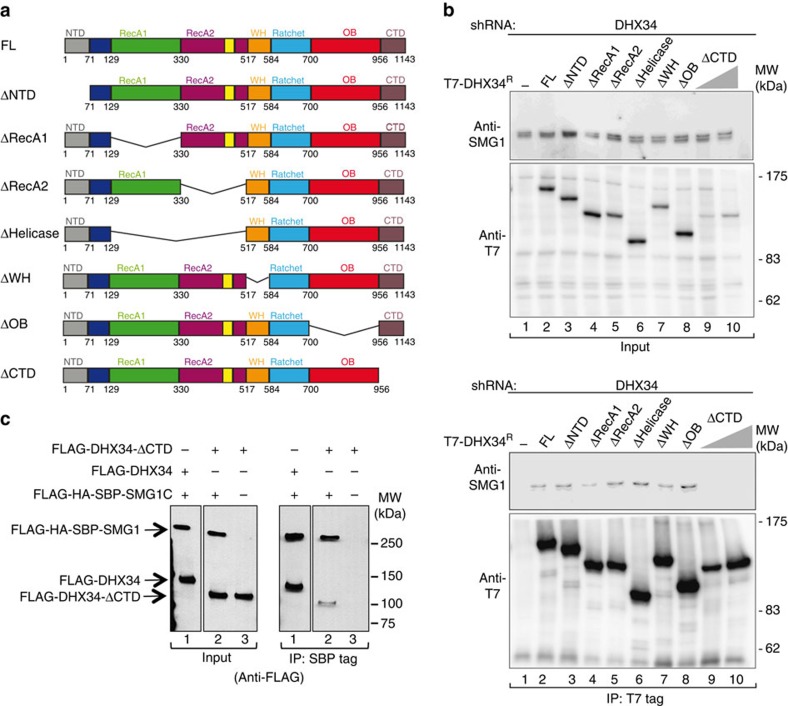
The CTD domain in DHX34 is the only domain required to bind SMG1 *in vivo* and *in vitro*. (**a**) Cartoon depicting the domain structure of DHX34 full length (FL) and mutants carrying deletions of individual domains. (**b**) HEK293T cells depleted of DHX34 with a specific shRNA were transfected with an shRNA-resistant version of full-length T7–DHX34 and DHX34 deletion mutants. Anti-T7 immunoprecipitates (20%, lower panel) were probed for the presence of SMG1. Inputs (0.5%) are shown on the upper panel. (**c**) Pull-down experiment testing the interaction of purified FLAG-DHX34 or mutant (ΔCTD) DHX34 to FLAG-SBP-HA-SMG1C. Proteins bound to Streptavidin Sepharose beads were eluted using biotin and analysed by SDS–PAGE and western blotting against the FLAG-tag in SMG1 and DHX34.

The relevance of the CTD domain in the recruitment of DHX34 to SMG1 *in vivo* was tested using a comprehensive collection of DHX34 deletion constructs, which comprise deletions of individual domains (Fig. 4a). The resulting constructs were transiently expressed as T7-tagged proteins in HEK293T cells that were depleted of endogenous DHX34 followed by immunoprecipitation (IP) with a T7-specific antibody and analysis of the amount of DHX34 and SMG1 in the input and IP fractions by western blot analysis, with anti-T7 and anti-SMG1 antibodies, respectively (Fig. 4b). The depletion of endogenous DHX34 and the levels of expression of the short hairpin RNA (shRNA)-resistant T7-tagged DHX34 constructs were determined using an antibody raised against the N terminus of DHX34 (Supplementary Fig. 6). Full-length DHX34 and most of the deleted versions of DHX34 retained their ability to bind endogenous SMG1 but, interestingly, only the ΔCTD construct showed a significant decrease in SMG1 binding. Although the ΔCTD construct was expressed at lower levels than the other deletion constructs (Fig. 4b upper panel), increasing the expression levels of the ΔCTD construct did not result in an interaction with SMG1 (Fig. 4b lanes 9 and 10 lower panel). To further support this finding, we tested the interaction of the ΔCTD mutant with SMG1C using purified proteins and SBP pull-down experiments (Fig. 4c). We found that whereas DHX34 interacted strongly with SMG1C, truncation of the C-terminal domain reduced binding to residual levels (Fig. 4c). Together, these results confirm the relevance of the SMG1–DHX34 interaction described by EM and demonstrate, in combination with the structural analysis of SMG1–DHX34, that the CTD is the major region in DHX34 strictly required to bind directly to SMG1.

### DHX34 binds UPF1 and SMG1 in separate sites

**Figure 5:**
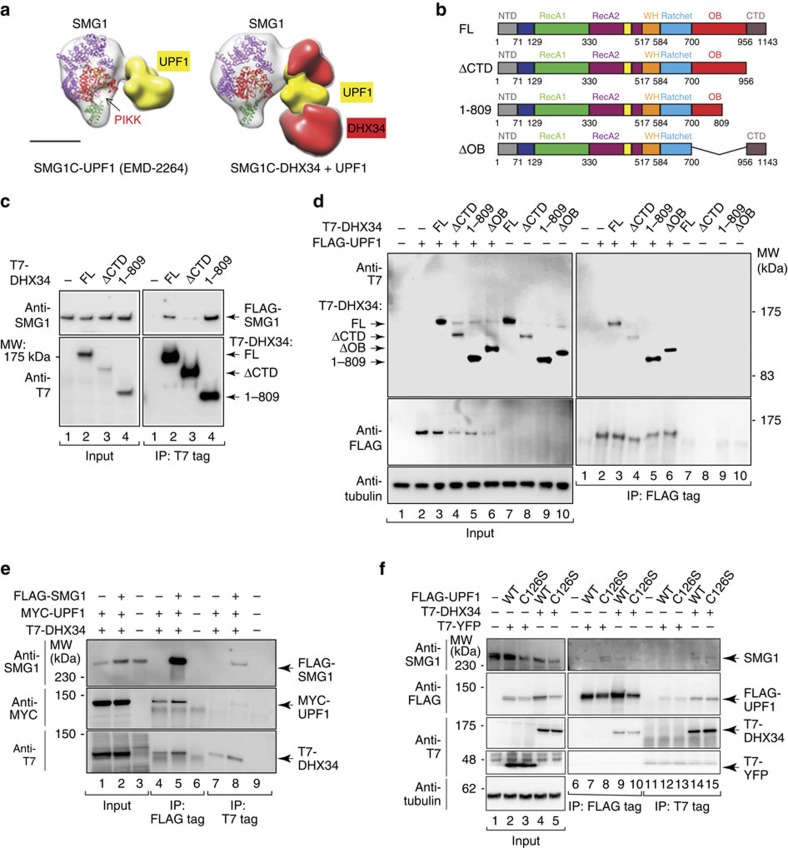
DHX34 can bind SMG1 via its CTD domain or via binding to UPF1. (**a**) Overlap between the structures of SMG1C–DHX34 and SMG1C-UPF1 (EMD-2264)^21^, revealing that the formation of a putative SMG1C-UPF1-DHX34 complex is structurally feasible. The putative location of the SMG1 kinase domain is indicated as PIKK in red. Scale bar, 5 nm. (**b**) Cartoon depicting the domain structure of DHX34 full-length (FL) and C-terminal deletion mutants. (**c**) HEK293T cells were transfected with full-length T7–DHX34 and DHX34 C-terminal deletion mutants shown in **b**. Inputs (0.5%) and anti-T7 immunoprecipitates (20%) were probed for the presence of SMG1. Expression of T7–DHX34 constructs was detected with an anti-DHX34 antibody. (**d**) Analysis of the binding of T7–DHX34 (FL and C-terminal deletion mutants) to FLAG-UPF1. HEK293T cells were transfected with full-length T7–DHX34 or DHX34 C-terminal deletion mutants and FLAG-UPF1. Inputs (0.5%) and anti-FLAG-immunoprecipitates (20%) were probed for the presence of the T7–DHX34 constructs. (**e**) Sequential co-IPs reveal a SMG1, UPF1 and DHX34 multi-protein complex. Cells expressing FLAG-SMG1, MYC-UPF1 and T7–DHX34 were subjected to anti-FLAG-IPs. FLAG-immunoprecipitates were then eluted using a FLAG peptide and immunoprecipitated using anti-T7 agarose. Inputs (0.5%) and anti-FLAG-IPs (20%) and anti-T7-IPs (10%) were probed with the indicated antibodies. (**f**) Purification of a tripartite containing SMG1, UPF1 and DHX34 as in **e** but with IP of FLAG-UPF1 (wild-type and C126S mutant), followed by IP of T7–DHX34 using anti-T7 agarose. Inputs (0.5%), anti-FLAG elutions (10%) and anti-T7-IPs (20%) were probed with the indicated antibodies.

Next, we set to define whether the binding sites for UPF1 and SMG1 in DHX34 overlap. We had previously identified the regions in DHX34 that are essential for the interaction with UPF1 at the N-terminal part of the protein, around the RecA domains^38^, a region we now describe as part of the globular core of DHX34. Therefore, it is reasonable to infer that the binding of UPF1 to DHX34 should not interfere with the binding of DHX34 to SMG1. When we modelled the putative overlap between UPF1 and DHX34 in the context of SMG1C, by superimposing the 3D structures of SMG1C–DHX34 (this work) and SMG1C–UPF1 (ref. 21), we found that there was not a significant clash between the two proteins, and thus an interaction of the CTD domain in DHX34 with SMG1, together with the simultaneous interaction of UPF1 to SMG1 and to the globular part of DHX34 is conceptually possible (Fig. 5a). We confirmed this biochemically by analysing a set of DHX34 deletion mutants (Fig. 5b) for their interaction with SMG1 (Fig. 5c) and with UPF1 (Fig. 5d), following similar strategies to those described above. The proteins analysed in this experiment comprise the DHX34 ΔCTD and ΔOB constructs analysed above (Fig. 4), but also included a truncation in DHX34, affecting the integrity of the OB-fold, DHX34 (1–809).

As described earlier, deletion of the CTD in DHX34 (ΔCTD) significantly reduced its interaction with SMG1 (Fig. 5c); however, we observed that a larger C-terminal deletion in DHX34 (1–809), which not only misses the CTD region but also lacks a large portion of the OB fold, binds to SMG1 significantly more efficiently than full-length DHX34. This raised the possibility that there could be an alternative mode of binding, whereby DHX34 could bind to SMG1 via binding to UPF1 acting independently of the CTD domain in cell lysates. For this, we analysed the interaction of all these four constructs with UPF1. We observed that all DHX34 constructs interacted with UPF1, including the deletion construct lacking CTD domain, which was expressed at a lower level, as shown above (Fig. 4b upper panel and Fig. 5d), confirming that UPF1 binds to the DHX34 core independently of the CTD domain. Of importance, we noticed that DHX34 (1–809) interacted significantly more strongly with UPF1 than the full-length protein (Fig. 5d), suggesting that the OB-fold negatively regulates UPF1 binding. This was confirmed by the stronger interaction with UPF1 displayed by ΔOB construct (Fig. 5d). No pull down of any DHX34 construct was observed if similar amounts of FLAG-GFP were immunoprecipitated (Supplementary Fig. 7a), ruling out the possibility that the effects observed for the DHX34 (1–809) are caused by the aggregation of the protein causing enhanced co-precipitation. These results were recapitulated *in vitro* by co-incubation of purified T7-DHX34 (full-length and the C-terminal deletions ΔCTD and 1–809) with recombinant UPF1 proteins, either with a purified FLAG-tagged full-length UPF1 or a truncated UPF1 construct (115–914). Again, an increased interaction of T7–DHX34 (1–809) with UPF1 was observed (Supplementary Fig. 7b, compare lanes 2 and 4 for full-length UPF1 and lanes 11 and 13 for the truncated UPF1 protein). In contrast, no difference in the affinity for UPF1 was observed for the full-length T7–DHX34 and the ΔCTD construct (Supplementary Fig. 7b, compare lanes 2 and 3 for full-length UPF1 and lanes 11 and 12 for the truncated UPF1 protein).

Taking into account (i) that the CTD is the only domain essential for recruitment to SMG1 (Fig. 4); (ii) that the integrity of the OB-fold is disrupted in the DHX34 (1–809) construct; (iii) that DHX34 (1–809) binds UPF1 much more tightly than wild-type DHX34; and (iv) most importantly, that UPF1 phosphorylation in the presence of DHX34 (1–809) is significantly affected compared with full-length DHX34 (see below), we interpret these results as an indication that DHX34 (1–809) is recruited indirectly to SMG1 through its enhanced interaction with UPF1.

To support this model, we tested whether SMG1C, DHX34 and UPF1 can form a tripartite complex, in which UPF1 can contribute to recruit DHX34 to SMG1 (Fig. 5e). For this purpose, FLAG-SMG1, T7-DHX34 and MYC-UPF1 were co-expressed in human cells and subjected to two sequential IPs: first, against the FLAG-tag (for SMG1), followed by FLAG peptide elution and then a second IP against the T7 tag (for DHX34). MYC-UPF1 was only recovered after the second IP if all three proteins, SMG1, UPF1 and DHX34, were co-expressed (Fig. 5e, compare lane 8 with lane 7 and 9), indicating that all three proteins are part of a multiprotein complex. To confirm that endogenous SMG1 can also be found in complex with DHX34 and UPF1, similar experiments were performed in cells by co-transfecting FLAG-UPF1 (either wild-type or a C126S UPF1 mutant) with T7–DHX34 or T7–YFP as a negative control. The C126S mutation in UPF1 prevents its interaction with UPF2 and consequently freezes the surveillance (SURF) complex^27^,^42^. SMG1 was only present in the second IP if FLAG-UPF1 (wild-type or C126S) and T7-DHX34 were co-expressed (compare lane 11 with lane 14 and 15). No SMG1 was co-purified if T7-YFP was co-expressed with FLAG-UPF1 (wild-type or C126S) (Fig. 5f, compare lane 12 and 13 with lane 14 and 15, respectively). Taken together, these findings demonstrate that the regions of DHX34 involved in UPF1 and SMG1 do not overlap completely, and that DHX34 can bind SMG1 and UPF1 simultaneously.

### The CTD domain supports UPF1 phosphorylation and NMD

**Figure 6:**
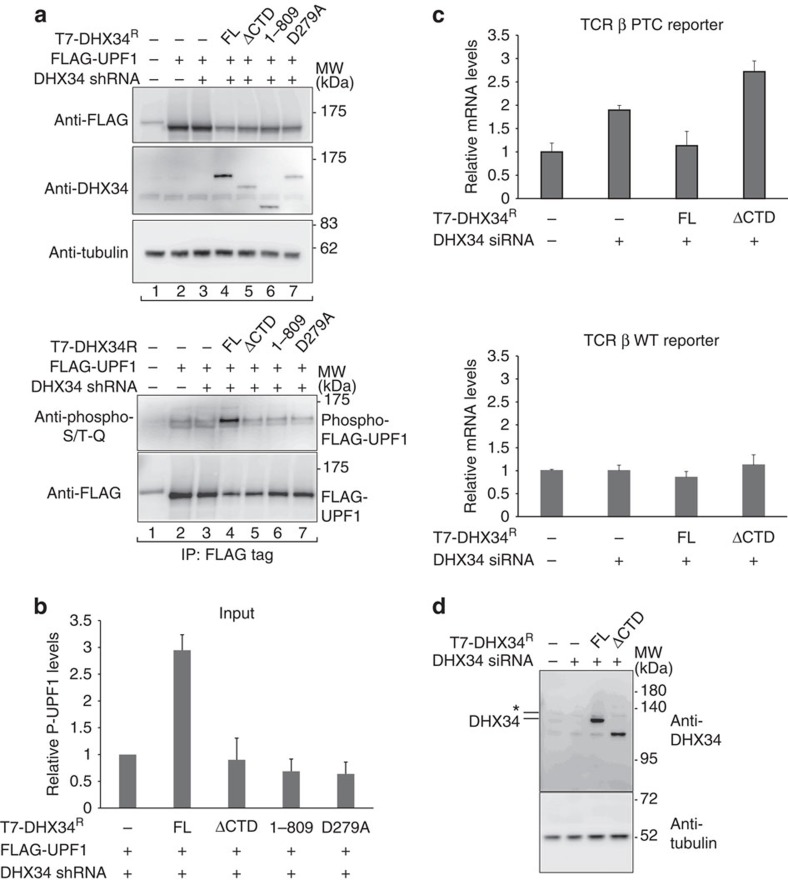
The CTD domain in DHX34 is required for efficient UPF1 phosphorylation and functional NMD. (**a**) HEK293T cells depleted of DHX34 with a specific shRNA or transfected with an shRNA targeting Luciferase (−) were co-transfected with FLAG-UPF1 and shRNA-resistant full-length (FL) T7–DHX34, a mutant lacking the CTD (ΔCTD), a mutant spanning residues 1–809 of DHX34 or a catalytic inactive (D279A) mutant. Phosphorylated UPF1 was detected with a phospho-(Ser/Thr) ATM/ATR substrate antibody. The phospho-FLAG-UPF1 signal was normalized to the levels of UPF1 recovered in the IP. (**b**) A quantification of the western blot signal from three independent experiments is shown. (**c**) NMD assay in HeLa cells transfected with a specific siRNA for DHX34 or a non-targeting control and two plasmids: one expressing an NMD reporter, TCR-β harbouring a PTC or a TCR-β WT reporter (lacking a PTC) and also including another plasmid expressing β-globin as a transfection control. Plasmids expressing siRNA-resistant full-length T7-tagged (FL) or mutant (ΔCTD) DHX34 proteins were included, as indicated. The T7 empty vector plasmid served as a control. The levels of wild-type or PTC-containing TCR-β reporters were analysed by qRT–PCR and normalized to those of β-globin mRNA used as a transfection control. Mean values±s.d. from three independent experiments are shown. (**d**) The expression of full-length or mutant T7–DHX34 was analysed by western blotting using an anti-DHX34 antibody. An antibody against tubulin served as a loading control. The lower band corresponds to DHX34, whereas an asterisk above indicates an unspecific band. For all panels, mean values±s.d. from three independent experiments are shown. Error bars represent s.d.

Next, we explored the functional consequences of abolishing the interaction of DHX34 and SMG1 on UPF1 phosphorylation. For this, full-length DHX34, ΔCTD, DHX34 (1–809) or D279A, a DHX34 ATPase-deficient mutant^38^, were co-expressed with FLAG-UPF1 after depletion of endogenous DHX34 using shRNA. The transcripts encoding DHX34 (full length or mutants) were resistant to the small interfering RNA (siRNA) because of the introduction of mutations that disrupt the mRNA–siRNA base pair interactions without changing the protein sequence. UPF1 was pulled down and the relative amount of phosphorylated and non-phosphorylated UPF1 was determined using phospho-specific antibodies, as previously described^38^. DHX34 strongly enhanced UPF1 phosphorylation (Fig. 6a,b) but, interestingly, the ratio of phosphorylated UPF1 was significantly reduced in both deletion mutants when compared with full-length DHX34, to similar levels as observed in cells lacking DHX34 or when using an ATPase-deficient mutant (Fig. 6a,b). Importantly, the total amount of UPF1 that binds to DHX34 (1–809) is substantially enhanced compared with full-length DHX34 (Fig. 5) and, therefore, the reduced phosphorylation of UPF1 is not due to a defect in the binding of DHX34 to UPF1. These results suggest that the direct interaction of DHX34 with SMG1 through the CTD is essential to stimulate phosphorylation. In addition, it strongly suggests that DHX34 can associate to SMG1 indirectly through UPF1, as revealed by the DHX34 1–809 deletion mutant; however, this is insufficient to activate UPF1 phosphorylation.

Next, we assayed for the requirement of the CTD and consequently of binding of DHX34 to SMG1, to confer NMD activity. For this, we used a previously described NMD complementation assay, using a well-characterized NMD reporter, the T-cell receptor (TCR)-β minigene harbouring a PTC, or a wild-type reporter (lacking a PTC) as a control^43^. Depleting DHX34 (70–80% reduction in mRNA levels) (Supplementary Table 1) caused the PTC-containing TCR-β reporter to increase by two-fold (Fig. 6c upper panel). In contrast, the expression levels of the corresponding wild-type mRNA of the TCR-β reporter did not change significantly (Fig. 6c lower panel). In cells lacking endogenous DHX34, expressing an siRNA-resistant version of full-length DHX34 restored NMD activity, as seen by the reduction in the expression levels of the TCR-β reporter (Fig. 6c left panel). By contrast, NMD activity was not restored in cells expressing an siRNA-resistant DHX34 lacking the CTD, where the PTC containing reporter was further stabilized (Fig. 6c). As the DHX34 ΔCTD mutant was expressed at similar levels as the full-length DHX34 (Fig. 6d), we concluded that the CTD domain is necessary for NMD.

## Discussion

UPF1 phosphorylation by the SMG1 kinase is a key event that defines the initiation of the NMD pathway and this is regulated by complex mechanisms requiring the function of several NMD factors^2^,^7^. UPF2 and UPF3 activate SMG1-mediated UPF1 phosphorylation in human cells, but additional factors have been described recently that contribute to regulate UPF1 phosphorylation, including the RuvBL1 and RuvBL2 ATPases^32^, and the RNA helicase DHX34 (refs 34, 38, 44). Here we have established several key aspects of how DHX34 functions to regulate NMD. First, we have found that the architecture of DHX34 is organized in two distinct structural regions, a helical core and a C-terminal region that protrudes from the core. Second, we determined that UPF1- and SMG1-binding sites in DHX34 are distinct and they are not mutually exclusive. The helicase core in DHX34 interacts with UPF1, whereas its CTD protrudes from the core and binds specifically to SMG1. Third, the CTD is the only domain in DHX34 required to recruit DHX34 to SMG1; however, results using the DHX34 (1–809) deletion mutant suggest that if the core of DHX34 is strongly bound to UPF1, DHX34 can be indirectly associated to SMG1 through UPF1. Fourth, the structure of the SMG1C–DHX34 complex reveals that CTD binds in the proximity of the SMG1 kinase domain. Fifth, SMG1, DHX34 and UPF1 can assemble in one complex. Finally, we define that the CTD domain, and therefore the specific interaction of DHX34 to SMG1, is necessary to activate UPF1 phosphorylation by SMG1 and also to support a functional NMD.

**Figure 7:**
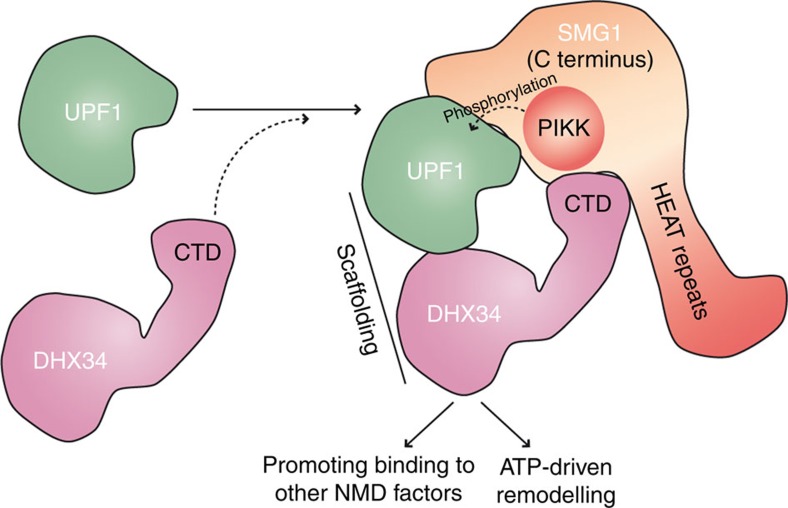
Molecular model for the function of DHX34 in NMD. DHX34 functions as a scaffold for UPF1 and SMG1, bringing the two proteins in the right orientation and placing UPF1 facing the SMG1 kinase domain. The CTD domain in DHX34 is essential for holding the SMG1-UPF1-DHX34 complex together. DHX34 could also contribute to UPF1 phosphorylation by facilitating the interaction of UPF1 with other NMD factors and the ATP-driven remodelling of the NMD complexes.

These results allow us to provide a molecular model for how DHX34 enhances UPF1 phosphorylation (Fig. 7). We propose that DHX34 functions as a scaffold for SMG1-mediated UPF1 phosphorylation, which brings together the kinase and its substrate. Importantly, DHX34 (1–809) binds UPF1 and SMG1 but it does not activate phosphorylation (Fig. 6); therefore, the function of DHX34 cannot be merely to increase the efficiency or the lifetime of the interaction between UPF1 and SMG1, to, in turn, enhance UPF1 phosphorylation. The structure of the SMG1C–UPF1 complex shows UPF1 in a well-defined orientation, facing SMG1 kinase domain, but the conformation of that complex was fixed with a mild cross-linking agent to help the structural analysis^21^. Instead, images of the SMG1C–UPF1 complex in the absence of cross-linking suggested some flexibility in the attachment between both proteins. The conformational flexibility of UPF1 when attached to SMG1C was clearly revealed by recent cryo-EM structures of the SMG1C–UPF1 complex^20^. Thus, we propose that DHX34 could possibly help to position UPF1 in the optimal orientation for phosphorylation, holding UPF1 close to the kinase domain, but also for interaction with other NMD factors. DHX34 promotes molecular transitions that mark NMD initiation such as binding of UPF2 and the EJC to UPF1 (ref. 38), whereas UPF2 and UPF3 activate the SMG1 kinase^27^,^42^. Thus, DHX34 could also contribute to facilitate the interaction of UPF1 with UPF2. This model would explain the requirement of the attachment of DHX34 to SMG1 through the CTD, to enhance phosphorylation and NMD.

A role of DHX34 to promote the interaction with other NMD factors *in vivo* would also rationalize why recombinant DHX34 does not stimulate UPF1 phosphorylation by SMG1 *in vitro* using purified SMG1 and UPF1 (ref. 38) but it is required for the activation of UPF1 phosphorylation in culture cells. Activation of SMG1 kinase activity *in vivo* requires the interaction of SMG1 with other factors^27^,^42^ and macromolecular changes promoting the transition from the Surveillance (SURF) to the Decay-inducing (DECID) complex^42^. ATP hydrolysis by DHX34 could possibly drive the remodelling of the NMD complexes required for UPF1 phosphorylation. The function of an RNA helicase as both a scaffold and an ATPase is not unique to DHX34 within the NMD pathway, as a similar situation is also found in UPF1 (ref. 2, 7). Moreover, our finding that the CTD truncated mutant interfered with mRNA degradation more strongly than DHX34 depletion itself in NMD complementation assays could be explained if the mutant DHX34 trapped UPF1 and/or another NMD factors in a non-productive complex.

The proposed model strongly parallels regulation of mTOR, another member of the PIKK family of kinases. Raptor was shown to regulate mTOR by a collection of findings: raptor forms a complex with mTOR; it plays an important but not obligatory role in mTOR-mediated phosphorylation of substrates; raptor forms a complex with both mTOR and substrates; and a deletion mutant of raptor that does not bind to mTOR inhibits the phosphorylation of mTOR substrates^45^,^46^. Raptor is now known to be part of mTORC1 complex, where it contributes to bring substrates to the active site. Our findings for DHX34 are reminiscent of raptor, supporting a role of DHX34 as a scaffold for UPF1 and SMG1 (Fig. 7). In addition, DHX34 would serve to also bring together other factors required to activate SMG1 and promote ATP-driven transitions required to promote UPF1 phosphorylation and NMD^38^.

## Methods

### Purification of proteins and protein complexes

FLAG-HA-SBP-SMG1C and SBP-GFP were produced and purified as described before^21^,^22^. Briefly, HEK293T cells were co-transfected with plasmids pEF_FLAG-HA-SBP-SMG1 (residues 1–3,657), pSR_Strep-HA-SMG9 (residues 2–520) and pSR_Strep-HA-SMG8 (residues 2–991) encoding SMG1, SMG8 and SMG9, respectively. SMG1C was purified using affinity chromatography by the SBP tag. The complex was eluted by incubation at 4 °C for 30 min with a buffer containing 2 mM biotin (Sigma-Aldrich). FLAG-DHX34 full-length and FLAG-DHX34 ΔCTD proteins were affinity purified as previously described^38^. HEK293T cells were grown in 10-cm plates and transfected using Lipofectamine 2000 according to the manufacturer's protocol. Two days after transfection, cells were lysed in IP buffer (10 mM Tris pH 8, 150 mM NaCl, 1 mM EGTA, 1% NP-40, 0.2% Na-Deoxycholate, Complete Protease Inhibitor (Roche), 1 mM dithiothreitol (DTT), 20 μg ml^−1^ RNase A (ThermoScientific). Following centrifugation for 10 min at 4 °C, supernatants were incubated with anti-FLAG resin (Sigma-Aldrich) at 4 °C for 2 h. Subsequently, beads were washed twice with IP buffer, with IP 1 M (IP buffer, supplemented with 1 M NaCl), with Buffer F (20 mM Tris-HCl pH 7.5, 1.2 mM EGTA, 250 mM Sucrose, 150 mM NaCl, 1% Triton X-100, 0.5% NP-40), Buffer F250 (Buffer F, supplemented with 250 mM LiCl), with Buffer D (20 mM HEPES-KOH pH 7.9, 100 mM KCl, 0.2 mM EDTA, 5% glycerol, 0.5% NP-40, 0.2% Na-Deoxycholate) and twice with Buffer D400 (Buffer D, supplemented with 400 mM KCl). Finally, the protein was eluted with IP Buffer supplemented with 3XFLAG-peptide at 250 μg ml^−1^. His-UPF1 (residues 115–914) was produced as previously described^21^. Full-length FLAG-UPF1 and T7-DHX34 (full-length or deletion constructs) were purified as described previously^38^ with the addition of 20 μg ml^−1^ RNase A (ThermoScientific) in the initial lysis buffer.

### cDNA constructs and immunoprecipitations

The plasmids pcG T7-DHX34, pcDNA3-3XFLAG-UPF1 and shRNA constructs have been described previously^38^. The DHX34 deletion mutants were cloned by PCR amplification, using the full-length DHX34 as a template. Primer sequences are available on request. For shRNA transfections, cells grown in six-well plates were transfected with 4 μg of plasmid shRNA pSuperpuro with Lipofectamine 2000 (Life Technologies), following manufacturer's instructions, and expanded 24 h later into selective media containing 0.75 μg Puromycin (Sigma-Aldrich). For co-IP experiments, cells were transfected 72 h later with 1 μg of shRNA, together with 2 μg of pcG T7-DHX34 constructs expressing either shRNA-resistant (^R^) wild-type, deletion constructs or empty vector controls and selected with medium containing Puromycin. Cells were washed 48 h later with 10 ml PBS and lysed in IP Buffer (10 mM Tris-HCl pH 8, 150 mM NaCl, 1 mM EGTA, 1% NP-40, 0.2% Na-Deoxycholate, Complete Protease Inhibitor (Roche), Phospho STOP (Roche), 1 mM DTT and 20 μg ml^−1^ RNase A). Cell lysates were cleared by centrifugation and incubated overnight with 40 μl Anti-T7 Tag Antibody agarose (69026, Novagen). The beads were washed five times and bound proteins were eluted with protein sample buffer and analysed by western blotting using an anti-SMG1 (ab30916, Abcam, dilution 1:1,000 or A300–393A, Bethyl, dilution 1:1,000) and monoclonal anti-T7 (69522, Novagen, dilution 1:5,000) antibodies. Expression levels of DHX34 constructs were determined using antibodies against an N-terminal peptide of human DHX34 and were described previously^38^ (dilution 1:1,000). An anti-tubulin antibody was used as a loading control (T5201, TUB 2.1 clone, Sigma-Aldrich, dilution 1:5,000). Secondary antibodies conjugated to horseradish peroxidase and ChemiGlow detection reagent were obtained from Bio-Rad and ProteinSimple, respectively. For FLAG-UPF1 and T7-DHX34 co-IPs, cells grown in six-well plates were transfected with 1 μg pcIneo-FLAG-UPF1 or pCMV-FLAG-GFP and 1 μg T7–DHX34 constructs, or the corresponding empty vector plasmids. Cells were expanded 24 h after and harvested 48 h after transfection. FLAG-UPF1 and FLAG-GFP were detected with anti-FLAG (F1804, M2 clone, Sigma-Aldrich, dilution 1:5,000) or anti-UPF1 (A300-036A, Bethyl, dilution 1:3,000) antibodies. For sequential co-IPs using FLAG-SMG1, MYC-UPF1 and T7–DHX34, 10 cm plates of HEK293T cells were transfected with 20 μg pCMV6-SMG1-MYC-FLAG (Origene), 5 μg pCMV-myc-UPF1 and 10 μg pcG T7-DHX34 or the relevant amounts of empty vector plasmids using Lipofectamine 2000 (Life Technologies) following manufacturer's instructions, expanded 24 h later. For sequential co-IP using T7-DHX34 and FLAG-UPF1, cells were transfected with 10 μg pcG T7-YFP or pcG T7-DHX34 and 10 μg pCDNA3-3XFLAG-UPF1 (wild-type or C126S mutant), or the corresponding empty vector plasmids. Forty-eight hours after transfections, cells were lysed in IP buffer as described above. Anti-FLAG-IPs were then eluted in 2 × bead volumes of IP buffer supplemented with 250 μg ml^−1^ FLAG peptide for 4 h at 4 °C, diluted with IP buffer and subjected to anti-T7 agarose IP. SMG1 was detected in the IP experiments using anti-SMG1 antibodies (A300–393A, Bethyl, dilution 1:1,000). MYC-UPF1 was detected with anti-c-myc (M4439, Sigma-Aldrich, dilution 1:5,000) and T7-tagged proteins with anti-T7 antibodies (69522, Novagen, dilution 1:5,000). For UPF1 phosphorylation experiments, cells were transfected 72 h later with 10 μg of shRNA, together with 20 μg of pcDNA3xFLAG-UPF1, as well as 20 μg of a plasmid expressing either shRNA-resistant (^R^) wild-type, deletion constructs or empty vector controls, selected with medium containing Puromycin and harvested for FLAG-IP 48 h later, as described above using anti-FLAG M2 affinity gel (A2220, Sigma-Aldrich). Protein complexes were analysed by western blotting and UPF1 phosphorylation was detected using Phospho-(Ser/Thr) ATM/ATR Substrate Antibody (2851, Cell Signaling, dilution 1:1,000) antibodies. FLAG-UPF1 levels in the IPs were detected with anti-FLAG (F1804, M2 clone, Sigma-Aldrich, dilution 1:5,000) antibodies. Signal were detected with the ImageQuant LAS 4000 system (GE Healthcare) and quantified using the ImageQuant TL Software (GE Healthcare). Uncropped scans of these experiments, corresponding to Figs 4, 5, 6, are provided as Supplementary Information (Supplementary Figs 8–11).

### NMD assays

Human TCR-β NMD reporters have been previously described^43^. Transfections of human HeLa cells were performed in six-well plates. Cells were initially transfected with a 50-pmol siRNAs using DharamFECT I (Dharmacon). Forty-eight hours later, cells were transfected using the Lipofectamine 2000 transfection reagent (Life Technologies) again with 50 pmol siRNAs together with a mixture of three plasmids: one expressing the NMD reporters (0.2 μg TCR-β reporters with or without PTC), one expressing the transfection control (0.2 μg β-globin WT)^47^ and a third (0.2 μg) expressing recombinant proteins (0.2 μg for DHX34 full-length, 0.4 μg for DHX34 ΔCTD) or the corresponding empty vector. The following siRNA were used: DHX34 (D-032233-02) and the non-targeting control (D-001206-13-20) (Dharmacon). Cells were harvested 48 h after the second round of transfection. Total RNA was isolated using the Qiagen RNeasy kit including DNase digestion RNA cleanup following the manufacturer's instruction. Complementary DNA was synthesized from 2 μg of total RNA using Transcriptor Universal cDNA Master (Roche) according to the manufacturer's instructions. Quantitative reverse trascriptase–PCR (qRT–PCR) was performed using the SYBR Green System (Roche). The expression of TCRβ reporter was normalized to the expression level of the transfection control (β-globin WT). The efficiency of the knockdown and the expression levels of the recombinant proteins were assessed by qRT–PCR and western blotting. For measuring the efficiency of depletion qRT–PCR was performed using the probe system (Roche) following normalization with three reference genes (*POL2RJ*, *MRIP* and *ACTB*). The sequences of the primers used for qRT–PCR and catalogue numbers for the Roche assays can be given on request. Uncropped scans of these experiments, corresponding to Fig. 6, are provided as Supplementary Information (Supplementary Fig. 11).

### *In vitro* pull-down experiments

To test the interaction between SMG1C and DHX34, FLAG-HA-SBP-SMG1C (17 nM, final concentration) was mixed with a three-fold molar excess of FLAG-DHX34 in SBP-binding buffer (20 mM Tris-HCl pH 7.5, 100 mM NaCl, 2.5 mM MgCl_2_, 0.5 mM DTT, 0.01% (v/v) Tween 20) in a final volume of 20 μl. The mixture was incubated while dialysing in SBP-binding buffer for 2 h at 4 °C and subsequently incubated with Streptavidin Sepharose High Performance Resin (GE Helthcare) for 2 h. The resin was washed three times with SBP-binding buffer and eluted with the same buffer supplemented with 2 mM Biotin. Interactions of UPF1 with SMG1C and DHX34 were tested by mixing His-UPF1 (115–914) (0.4 μM, final concentration) and a two-fold molar excess of FLAG-DHX34 and/or 15 nM FLAG-HA-SBP-SMG1C in Ni-NTA binding buffer (40 mM Tris-HCl pH 7.5, 200 mM NaCl, 10% (v/v) glycerol, 20 mM imidazole, 1 mM MgCl_2_, 1 μM ZnCl_2_, 2 mM DTT, 0.1% (v/v) NP-40) in 30 μl. After 20 min of incubation at 25 °C, the mixture was incubated with Ni-NTA Agarose resin (Qiagen) for 30 min at 4 °C, washed twice with Ni-NTA-binding buffer, once with Ni-NTA-binding buffer containing 50 mM imidazole and eluted with the same buffer containing 500 mM imidazole. Proteins were analysed by SDS–PAGE and Oriole Fluorescent Stain (Bio-Rad) and/or western blotting using anti-FLAG (M2 clone, Sigma-Aldrich, catalogue number F1804; dilution, 1:500) and/or anti-His antibodies (Sigma-Aldrich, catalogue number H1029; dilution 1:5,000). Pull downs testing the interaction between T7-DHX34 constructs and UPF1 were performed as described previously^38^.

### EM and image processing

Purified DHX34 and SMG1C-DHX34 complexes were adsorbed on carbon-coated grids, stained using 1% uranyl formate and observed using a JEOL-1230 operated at 100 kV. SMG1C-DHX34 complexes were assembled by mixing a 1.5:1 molar ratio of DXH34 and SMG1C, with the excess of DHX34 intended to favour major occupancy of SMG1C. The SMG1C-DHX34 complex was fixed by incubating the complex for 10 min in the presence of 0.02% glutaraldehyde at 25 °C. Images of single molecules were obtained automatically using a TVIPS F416 CMOS and a final magnification of 54926. Contrast transfer function was corrected using BSOFT^48^. A total of 50,193 images of DHX34 and 60,070 images of SMG1C–DHX34 at 5.68 Å per pixel were extracted from the micrographs automatically, and then classified and averaged using methods implemented in XMIPP^49^. Image classification was used to remove those images that did not correspond to molecule images but were incorrectly selected by the unsupervised automatic particle picking. In addition, for those images obtained after mixing DHX34 and SMG1C, a strategy based on a combination of two-dimensional and 3D classification methods was performed to allow the discrimination of images derived from SMG1C, DHX34 or SMG1C–DHX34 complexes (see Supplementary Fig. 4 for details). After classification of DHX34 images, 17,751 were identified as potential dimers and 12,316 as monomers (Supplementary Fig. 2). Only the monomeric species were used for 3D refinement (Supplementary Fig. 3). For SMG1C–DHX34, 13,080 images were unambiguously identified to correspond to the complex (Supplementary Fig. 4) and only these were then used for 3D refinement (Supplementary Fig. 5). Templates for angular refinement were obtained using the volume generator from EMAN2 (ref. 50) using as input unbiased reference-free averages obtained for each of the data sets and the images were subsequently refined using XMIPP^49^ and EMAN^50^ (Supplementary Figs 3 and 5). The resolution of the structures was estimated using the Fourier shell correlation method and a 0.5 correlation coefficient as 24.23 and 20.89 Å for DHX34 and DHX34–SMG1C, respectively (Supplementary Figs 3 and 5). Fitting of atomic structures into the EM maps was performed using UCSF Chimera^51^. The handedness of the reconstructions was determined by comparing with the published structure of SMG1C and the hand determined for DHX34 within the SMG1C–DHX34 complex was then applied to DHX34. Atomic structures used for the SMG1 model were the crystal structures of DNA-PKcs (PDB ID 3KGV)^19^ and mTOR (PDB ID 4JSP)^18^. For DHX34, the core fitted into the EM density with cross-correlation values above 0.90.

## Additional information

**Accession codes:** The EM maps of DHX34 and SMG1C–DHX34 have been deposited in the EM database (EMD-3279 and EMD-3278 for DHX34 and SMG1C–DHX34, respectively).

**How to cite this article:** Melero, R. *et al*. The RNA helicase DHX34 functions as a scaffold for SMG1-mediated UPF1 phosphorylation. *Nat. Commun.* 7:10585 doi: 10.1038/ncomms10585 (2016).

## Supplementary Material

Supplementary Information Supplementary Figures 1-11, Supplementary Table 1 and Supplementary References

Supplementary Movie 1 Movie showing several views of DHX34 and SMG1C-DHX34, represented as in Figure 1 and 3. The EM structures are shown as a transparent density where crystal structures were fitted. The model for SMG1C-DHX34 in the movie was built by superimposing the structure of DHX34 when bound to SMG1C, with the published structure of SMG1C (EMD-2663).
