# Supplementary material for: The RNA helicase DHX34 functions as a scaffold for SMG1-mediated UPF1 phosphorylation
Source: Nat Commun. 2016 Feb 4;7:10585. doi: 10.1038/ncomms10585 (PMC4743010; doi:10.1038/ncomms10585)
Supplement: Supplementary Information — Supplementary Figures 1-11, Supplementary Table 1 and Supplementary References [file ncomms10585-s1.pdf]

Supplementary Information

Supplementary Figures

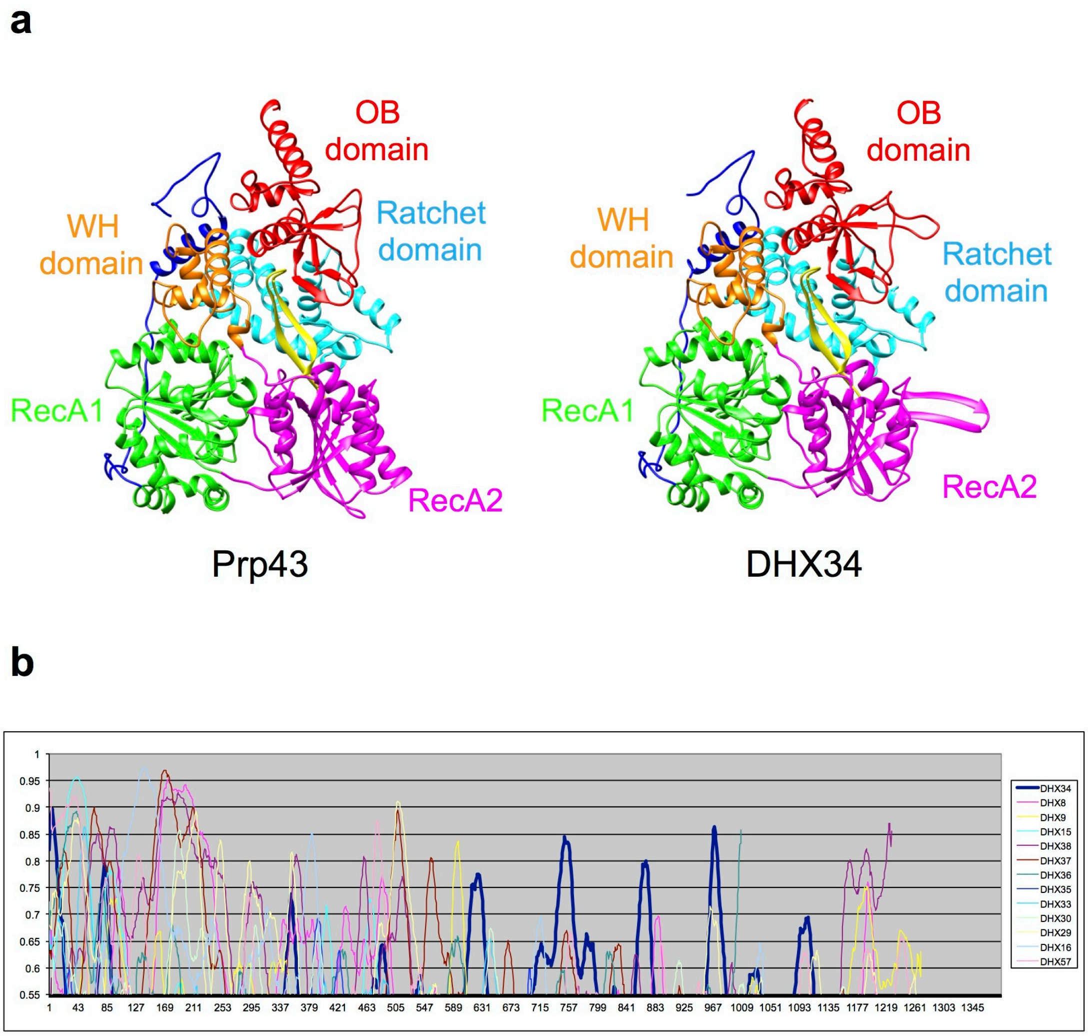

Supplementary Figure 1 | Structural prediction of DHX34. (**a**) Structural prediction of DHX34 based on the structure of Prp43, and obtained using PHYRE2[1]. Domains in both proteins are colored using the same color code. (**b**) The RONN (Regional Order Neural Network) software program[2], version 3.2 (kindly donated by authors), was used under a local customized Linux installation for the prediction of the natively disordered regions of 35 DHX protein sequences[3]. In order to compare the different sequence

predictions we displayed the RONN results with the command-line graphing program gnuplot embedded into a user-made Linux script. Each protein is represented with a different color. DHX34 is represented using a thicker dark blue line. The predictions suggest that disorder propensity in DHX34 accumulates at the C-terminal end of the protein.

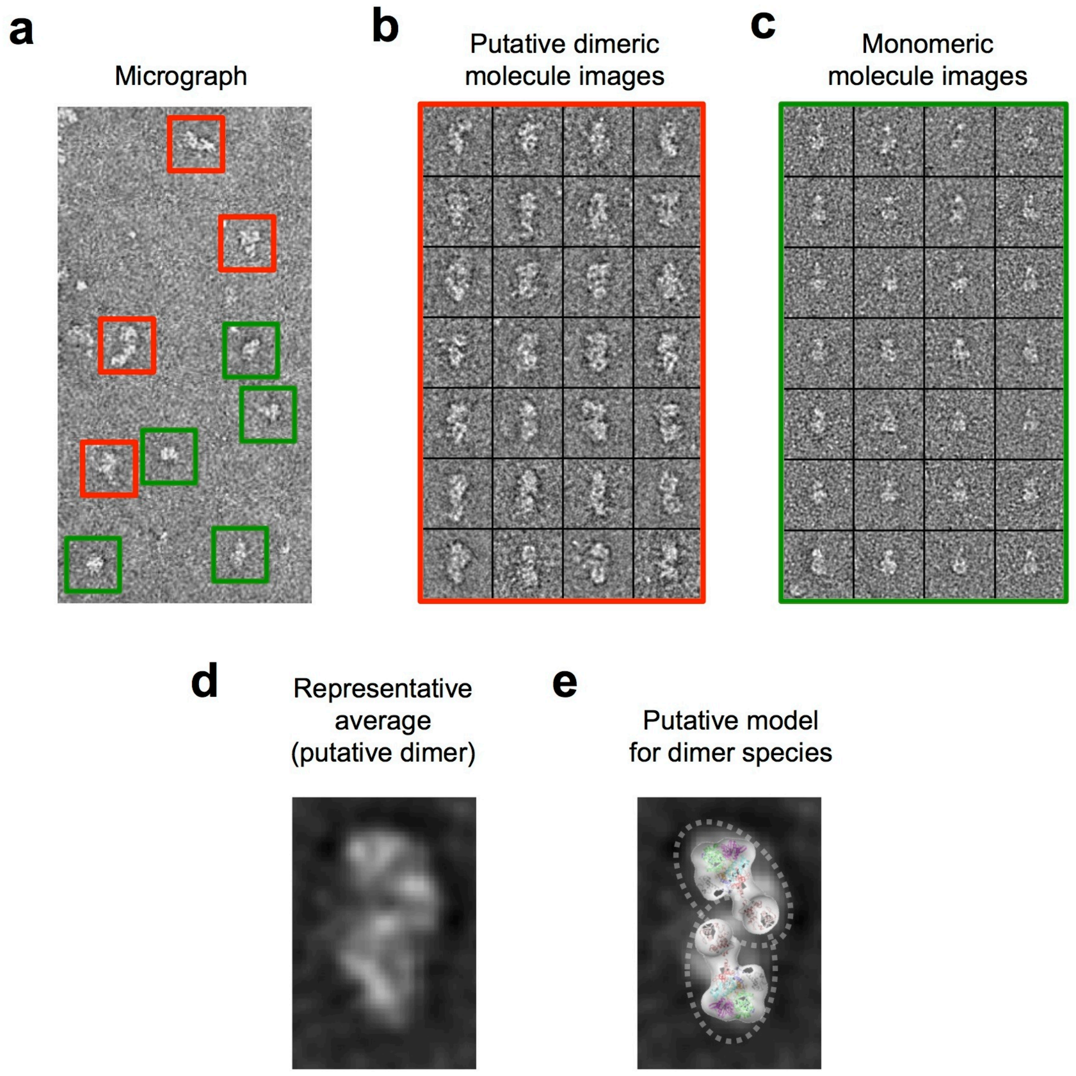

Supplementary Figure 2 | Electron microscopy of purified DHX34. (a) Representative micrograph obtained for DHX34, where several images of individual molecules are indicated under green squares (for monomers), and red squares (for the

putative dimers). (**b**) Selected images of molecules of DHX34 larger species, interpreted as possible dimers. (**c**) Selected images of molecules of DHX34 monomers. (**d**) Representative reference-free average of putative dimers. (**e**) The average of the putative dimers shown in (d) was used to model the possible configuration of the interactions within the dimers.

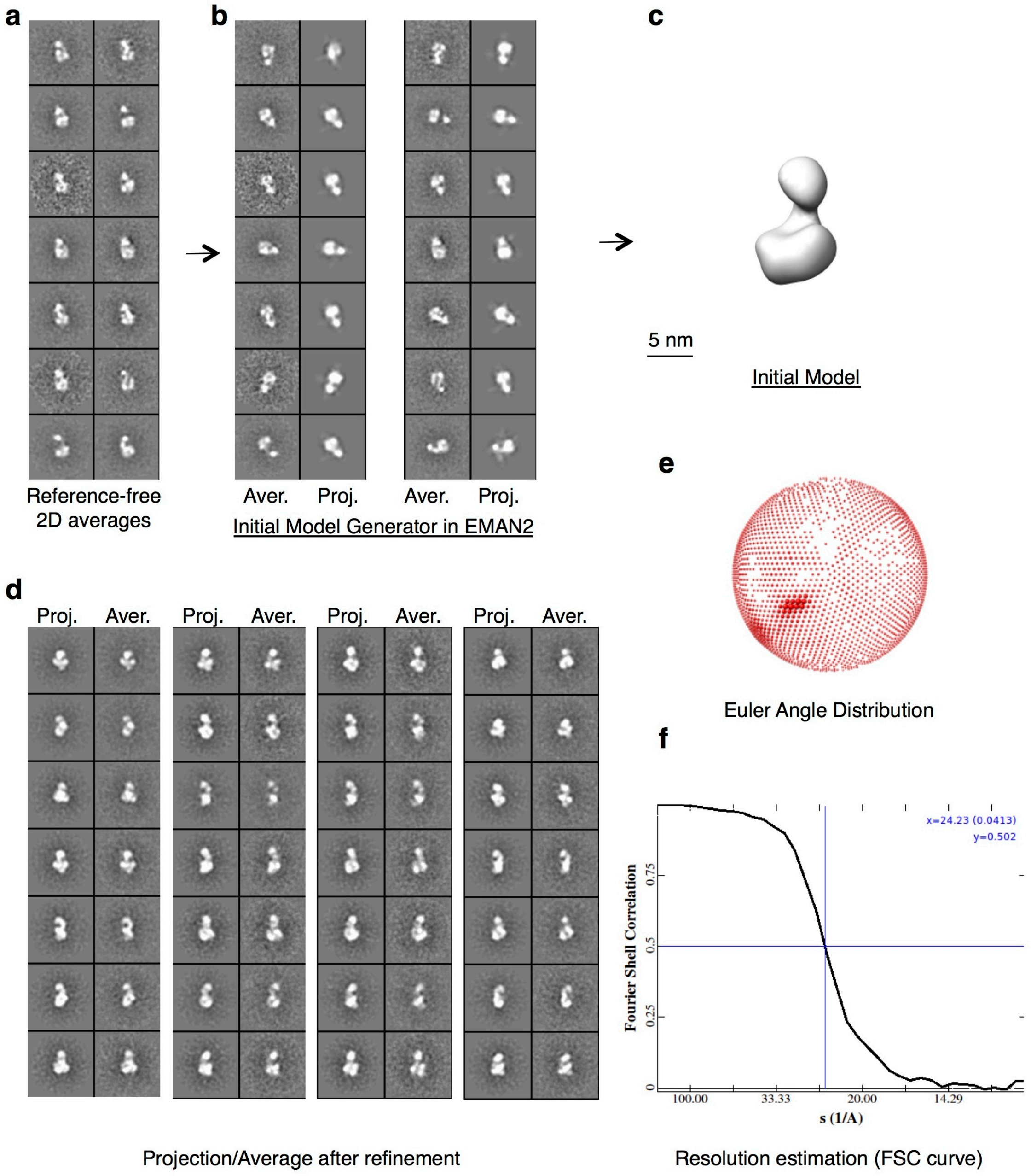

Supplementary Figure 3 | Image processing and 3D structure of DHX34. (**a**) Representative reference-free averages of DHX34 monomers. Averages of monomers are also shown in Fig. 1c. (**b**) A 3D template for angular refinement was obtained without any initial bias, using reference-free averages of DHX34 and the volume generator in EMAN2 (command used: e2initialmodel.py)[4]. The method tentatively assigns a certain orientation (Euler angles) to each reference-free average provided, and a 3D average volume is computed. Inspecting the similarity between projections of the 3D template in several orientations and the reference-free averages monitors the quality of the prediction. (**c**) Initial 3D template obtained for DHX34 using e2initialmodel.py in EMAN2[4]. (**d**) Comparison between some of the theoretical projections of the final 3D structure of DHX34, and the 2D averages of all particles classified within a certain orientation after angular refinement. (**e**) Euler angles distribution of DHX34 images in the dataset after refinement. The orientations found in the data set are represented as red dots within a sphere, as implemented in XMIPP (http://xmipp.cnb.csic.es/twiki/bin/view/Xmipp/WebHome). (**f**) The resolution of the structures was estimated using the Fourier Shell Correlation (FSC) method and a 0.5 correlation coefficient as 24.23 Å.

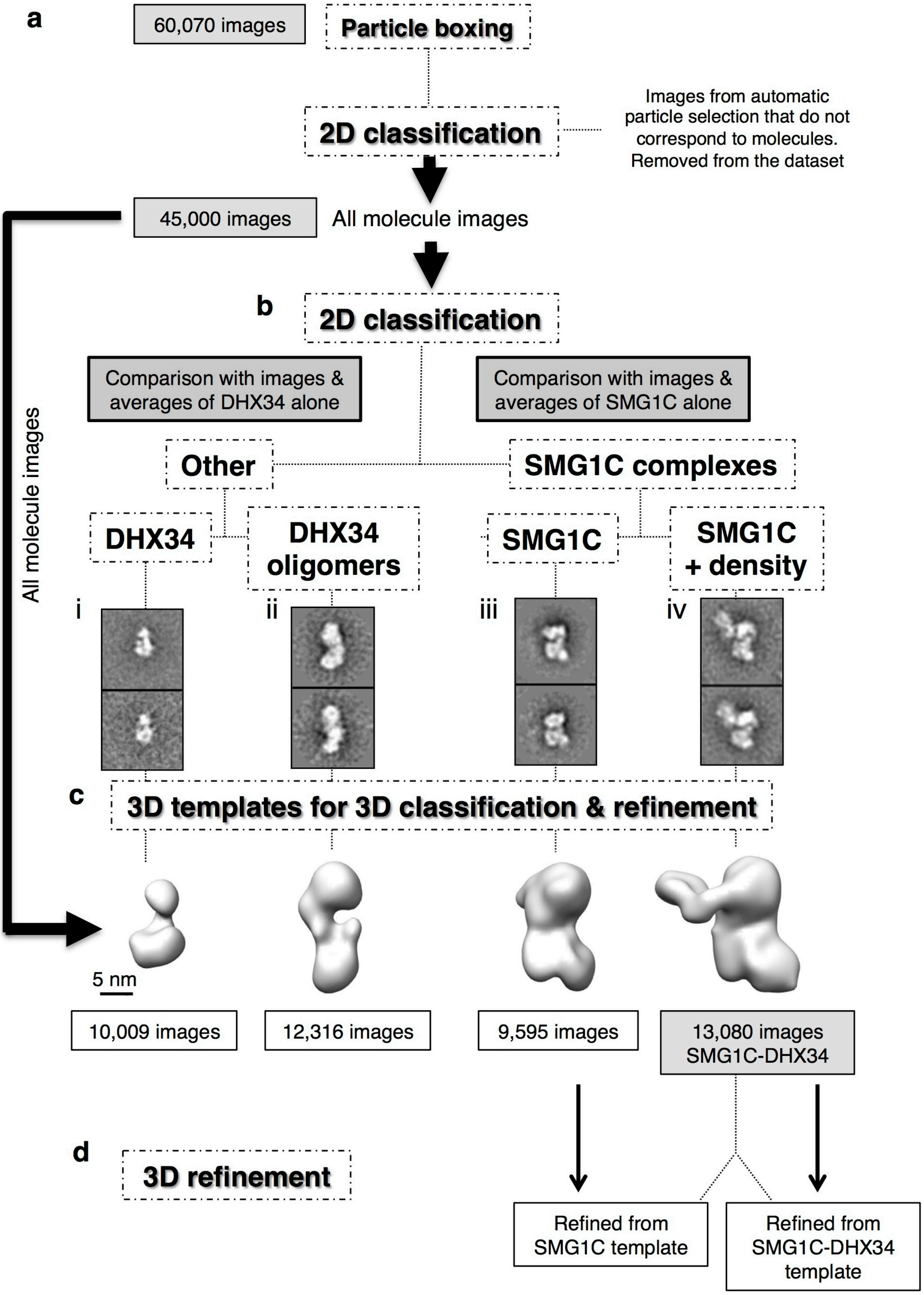

Supplementary Figure 4 | Image processing strategy to classify those images corresponding to DHX34, SMG1C and SMG1C-DHX34 after mixing DHX34 and

SMG1C. The mixing of DHX34 and SMG1C was expected to generate four main species, free DHX34 (monomers and oligomers seen in the analysis of DHX34 alone), free SMG1C, and the SMG1C-DHX34 complex. An image classification strategy was devised to identify the images corresponding to each of these species in the micrographs. The strategy relied on the knowledge of the images generated by DHX34 and SMG1C alone, analyzed before mixing. (**a**) The whole data set (60,070 images) was subjected to 2D reference-free classification methods. A first round of classification was used to remove those images that did not correspond to molecule images but were incorrectly selected by the unsupervised automatic particle picking. These were easily identified, as they appeared as noisy and featureless average images. 45,000 images assigned to classes showing features, therefore corresponding to images of molecules, were then split and classified again (**b**). Subsequently, the new 2D averages obtained were first compared with those averages obtained for DHX34 and SMG1C as controls. Images of DHX34, either monomers or dimers, were very different to those of SMG1C, and similar to the averages obtained for DHX34 alone (Fig. 1 and Supplementary Fig. 2). These images could then be readily identified and split from the data set. Similarly, images corresponding to SMG1C, showing or not, an additional density attached, were clearly identified by comparison with our previous work[5] and these were split to generate a new independent dataset. (**c**) Averages assigned to either DHX34 monomers, oligomers, SMG1C or SMG1C-DHX34 were used to generate unbiased low-resolution 3D templates for refinement and classification using the volume generator from EMAN2[4], Maximum-likelihood 3D classification methods as implemented in XMIPP[6], and using these four templates as seeds, were employed to re-classify the 45,000 images. Following this strategy, 13,080 images were sub-classified in a group of images

similar to SMG1C but showing a density attached to SMG1C, which was considered to correspond to the SMG1C-DHX34 complex. (**d**) Images of SMG1C-DHX34 were then refined using as initial reference a low-pass filtered version of the SMG1C or SMG1C-DHX34 templates, using EMAN. Supporting the robustness of the methodology applied, images classified as SMG1C-DHX34 generated a similar result in both refinements, using either SMG1C alone or SMG1C-DHX34 as templates. Additionally, although the information about DHX34 was never used for the refinement of the images of SMG1C-DHX34 at any stage, the 3D structure of DHX34 bound to SMG1C is closely similar to that obtained from DHX34 alone (Fig. 3c), supporting the classification strategy used.

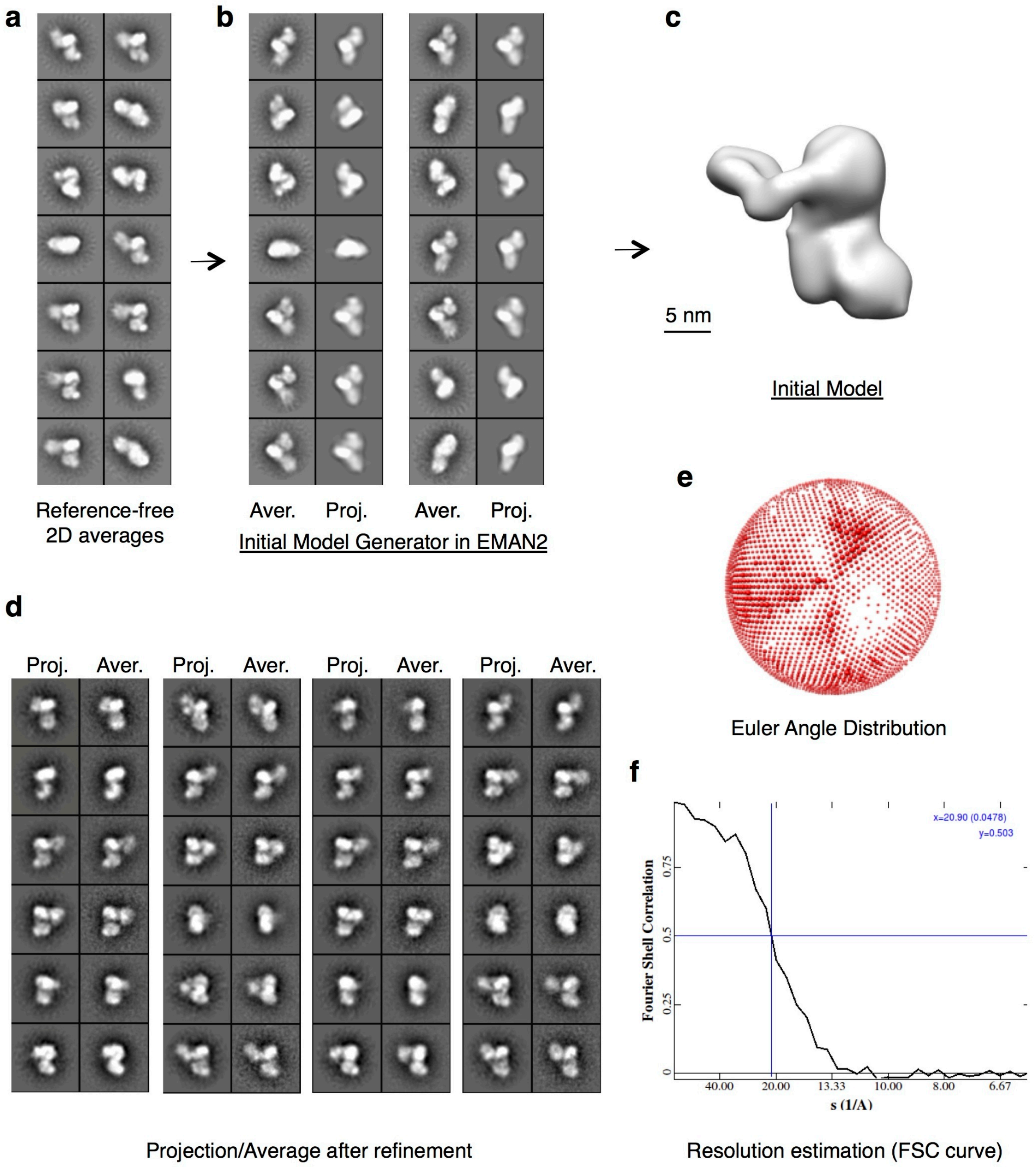

Supplementary Figure 5 | Image processing and 3D structure of SMG1C-DHX34. (**a**) Representative reference-free averages of SMG1C-DHX34 complexes. Averages of these complexes are also shown in Fig. 3a. (**b**) A 3D template for angular refinement was obtained without any initial bias, using reference-free averages of SMG1C-DHX34 (images of the complex split from the total dataset after the image classification strategy described in Supplementary Fig. 4), and the volume generator in EMAN2 (command

used: e2initialmodel.py)[4]. The method tentatively assigns a certain orientation (Euler angles) to each reference-free average provided, and a 3D average volume is computed. Inspecting the similarity between projections of the 3D template in several orientations and the reference-free averages monitors the quality of the prediction. (**c**) Initial 3D template obtained for SMG1C-DHX34 using e2initialmodel.py in EMAN2[4]. (**d**) Comparison between some of the theoretical projections of the final 3D structure of SMG1C-DHX34, and the 2D averages of all particles classified within a certain orientation after angular refinement. (**e**) Euler angles distribution of SMG1C-DHX34 images in the dataset after refinement. The orientations found in the data set are represented as red dots within a sphere, as implemented in XMIPP (http://xmipp.cnb.csic.es/twiki/bin/view/Xmipp/WebHome). (**f**) The resolution of the structures was estimated using the Fourier Shell Correlation (FSC) method and a 0.5 correlation coefficient as 20.89 Å.

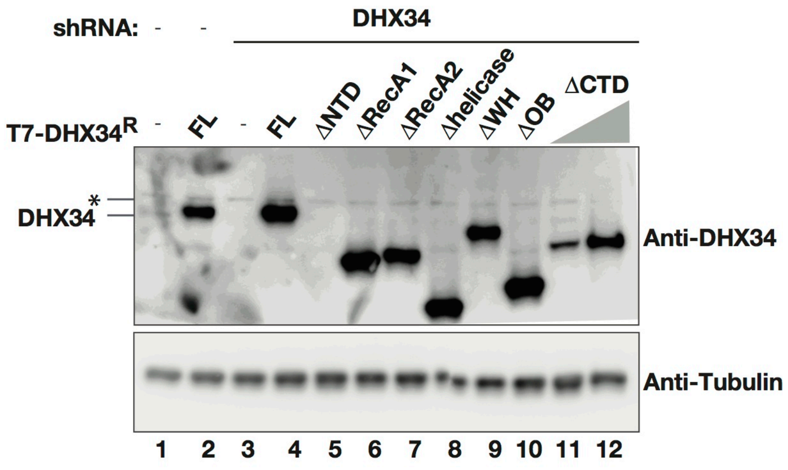

Supplementary Figure 6 | Western Blot showing the depletion of endogenous DHX34 and expression levels of T7-DHX34 constructs (full-length (FL) and deletion constructs) used in the T7-Immunoprecipitation experiments by probing with an antibody directed against an N-terminal peptide of DHX34. Therefore the ΔNTD construct is not detected. The lower band corresponds to DHX34, whereas an asterisk above indicates an unspecific band.

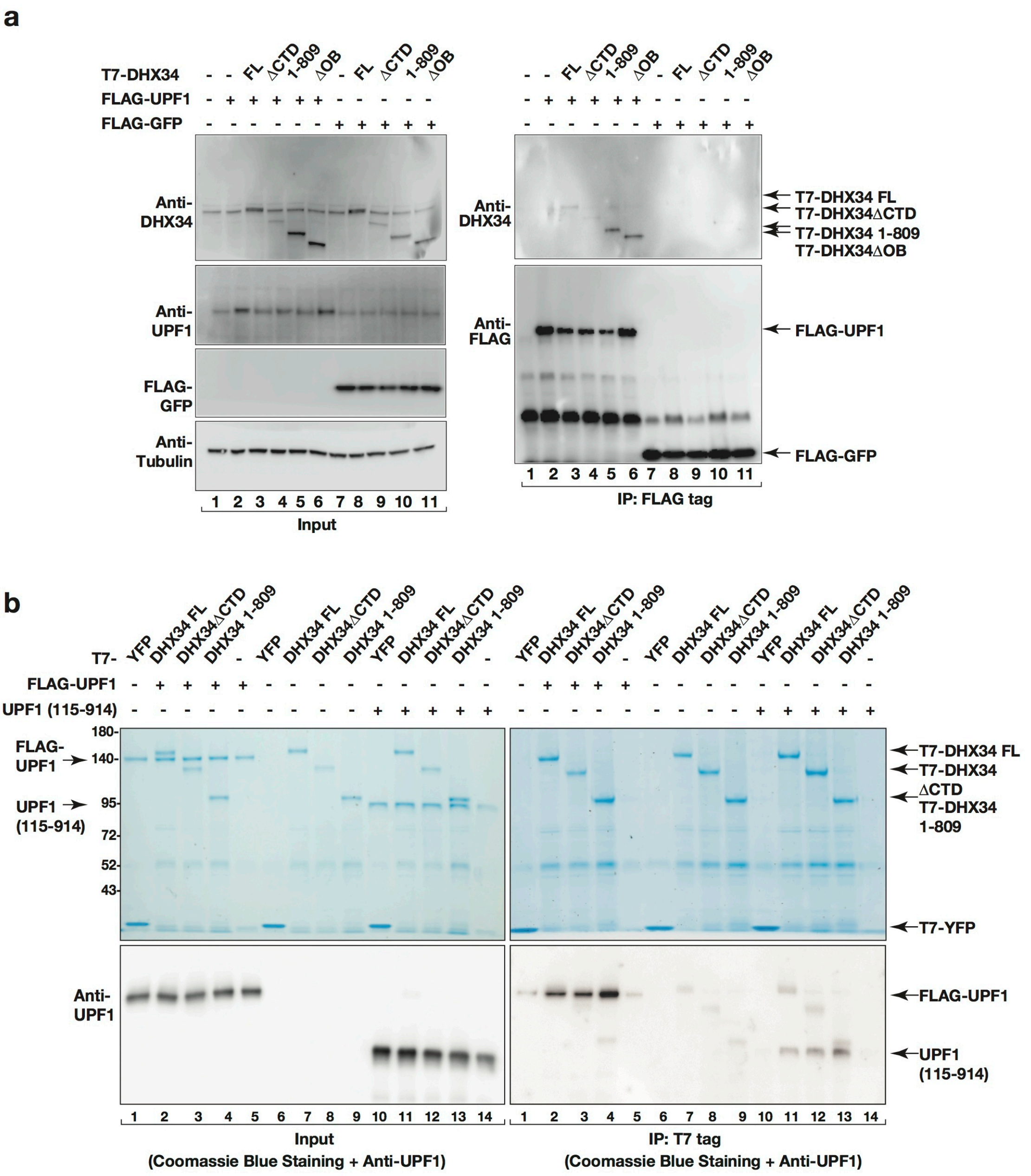

Supplementary Figure 7 | (**a**) Immunoprecipitation of transiently transfected T7-DHX34 (full-length or deletion mutants) and FLAG-UPF1 or FLAG-GFP from HEK293T cells. Inputs (0.5%) and anti-FLAG-IPs (20%) were subjected to Western Blot analysis using the indicated antibodies. (**b**) Interaction between purified T7-DHX34 (full-length or deletion mutants) and purified FLAG-UPF1 (full-length) or

recombinant truncated UPF1 (115-914), which were mixed in a 1:1 ratio and pulled down using T7 beads. Inputs (5%) and pulldown fractions (20%) were analyzed after SDS-PAGE by Coomassie Blue Staining. UPF1 was detected by Western Blotting using anti-UPF1 antibodies.

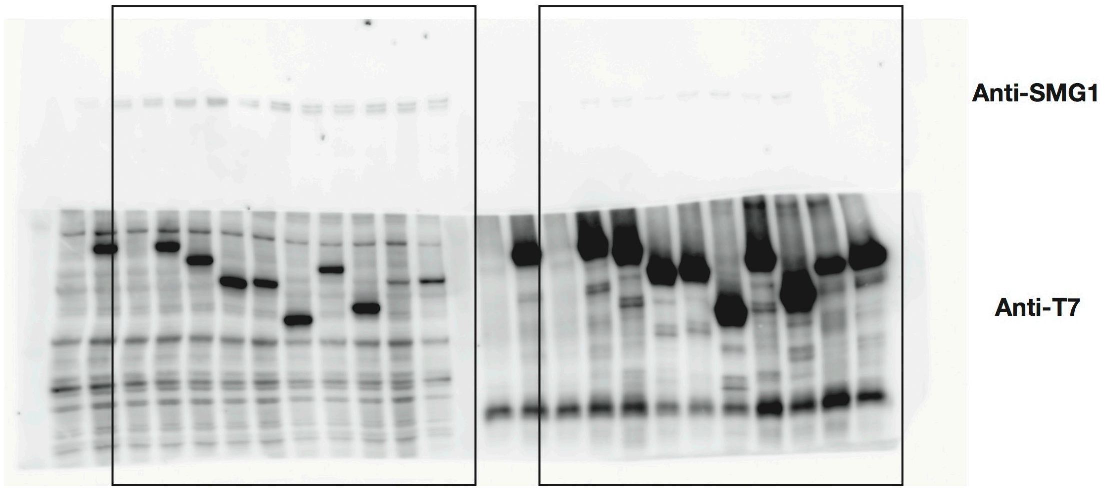

Supplementary Figure 8 | Uncropped scan Figure 4b.

Figure 5 c

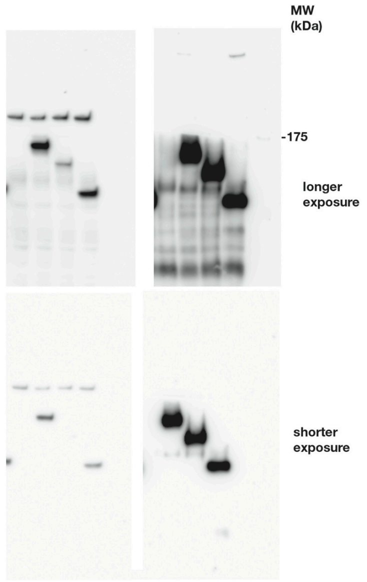

Figure 5 d

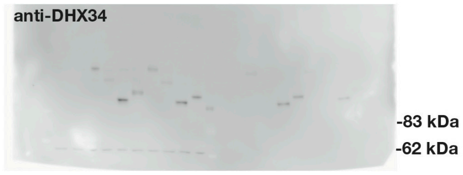

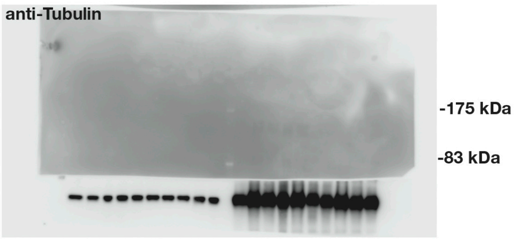

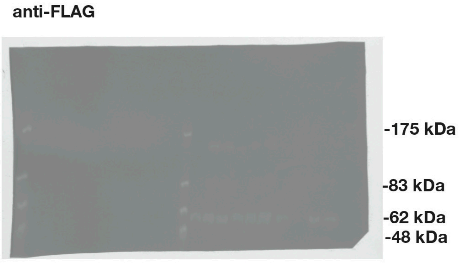

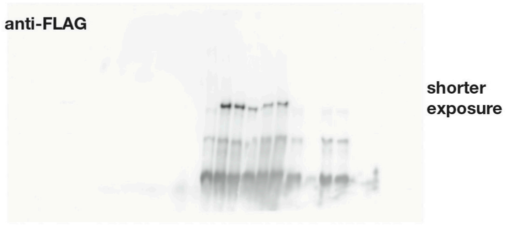

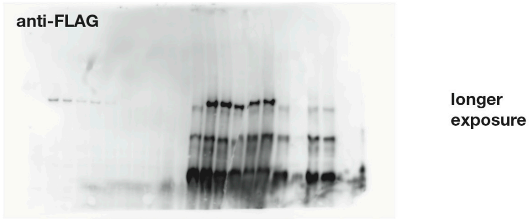

Supplementary Figure 9 | Uncropped scan Figure 5c and 5d.

Uncropped panel Figure 5e

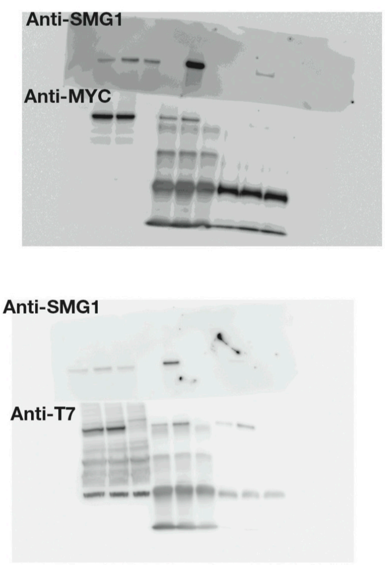

Uncropped panel Figure 5f

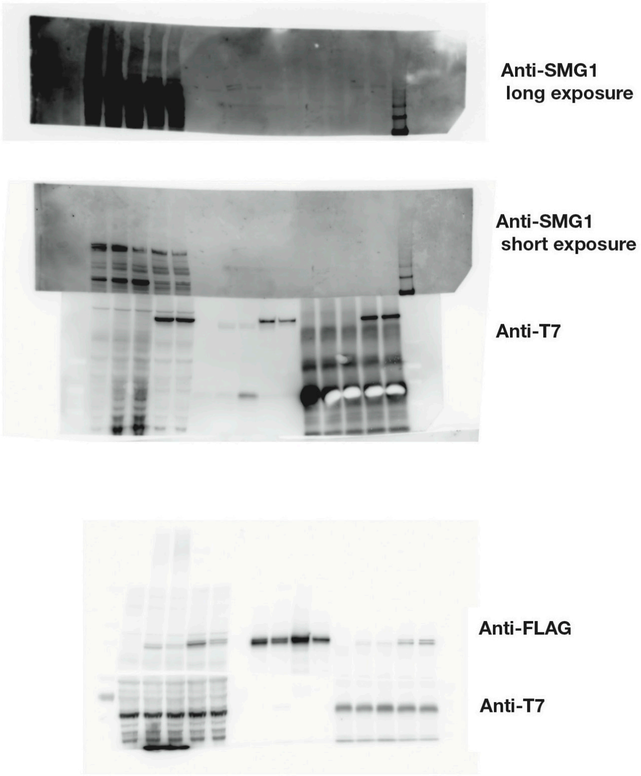

Supplementary Figure 10 | Uncropped scan Figure 5e and 5f.

Figure 6a

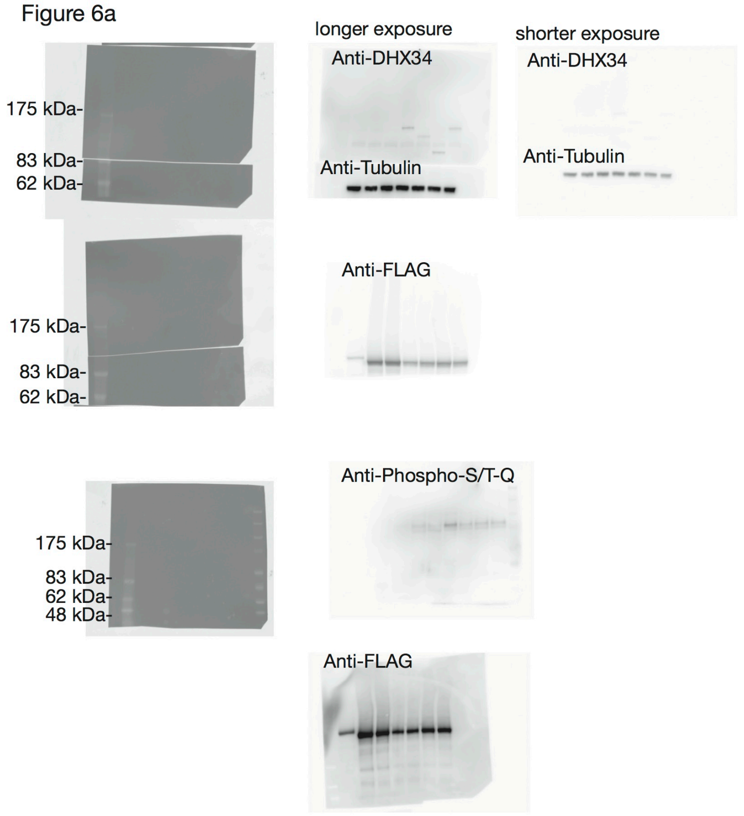

Figure 6d

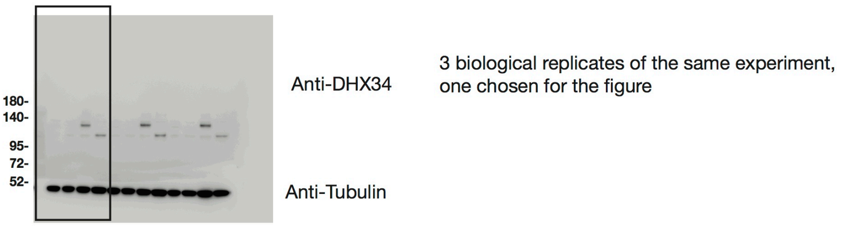

Supplementary Figure 11. Uncropped scan Figure 6a and 6d.

DHX34 mRNA levels in NMD complementation assay using TCR β PTC reporter

siRNA	T7 construct	DHX34 mRNA levels ± Stev
Non-targeting	Empty vector	1 ± 0.1
DHX34	Empty vector	0.21±0.02
DHX34	T7-DHX34-FL	14.9 ±3.0
DHX34	T7-DHX34ΔCTD	27.4 ±2.7

DHX34 mRNA levels in NMD complementation assay using TCR β WT reporter

siRNA	T7 construct	DHX34 mRNA levels ± StDev
Non-targeting	Empty vector	1 ± 0.04
DHX34	Empty vector	0.26 ± 0.03
DHX34	T7-DHX34-FL	12.9 ± 1.3
DHX34	T7-DHX34ΔCTD	20 ± 2.9

Supplementary Table I

Depletion and mRNA expression levels of DHX34 in NMD complementation assay shown in Figure 6. Average levels determined by quantitative qRT-PCR from three independent experiments ± standard deviations are presented.

Supplementary References

1 Kelley, L. A. & Sternberg, M. J. Protein structure prediction on the Web: a case study using the Phyre server. *Nat Protoc* **4**, 363-371 (2009).

2 Yang, Z. R., Thomson, R., McNeil, P. & Esnouf, R. M. RONN: the bio-basis function neural network technique applied to the detection of natively disordered regions in proteins. *Bioinformatics* **21**, 3369-3376 (2005).

3 Abdelhaleem, M., Maltais, L. & Wain, H. The human DDX and DHX gene families of putative RNA helicases. *Genomics* **81**, 618-622 (2003).

4 Tang, G. *et al.* EMAN2: an extensible image processing suite for electron microscopy. *Journal of structural biology* **157**, 38-46, (2007).

5 Melero, R. *et al.* Structures of SMG1-UPFs complexes: SMG1 contributes to regulate UPF2-dependent activation of UPF1 in NMD. *Structure* **22**, 1105-1119 (2014).

6 Sorzano, C. O. *et al.* XMIPP: a new generation of an open-source image processing package for electron microscopy. *Journal of structural biology* **148**, 194-204 (2004).
